# A dissipation-induced superradiant transition in a strontium cavity-QED system

**DOI:** 10.1126/sciadv.adu5799

**Published:** 2025-04-25

**Authors:** Eric Yilun Song, Diego Barberena, Dylan J. Young, Edwin Chaparro, Anjun Chu, Sanaa Agarwal, Zhijing Niu, Jeremy T. Young, Ana Maria Rey, James K. Thompson

**Affiliations:** ^1^ JILA, NIST, and Department of Physics, University of Colorado, Boulder, CO, USA.; ^2^ T.C.M. Group, Cavendish Laboratory, University of Cambridge, J.J. Thomson Avenue, Cambridge CB3 0HE, UK.; ^3^ Center for Theory of Quantum Matter, University of Colorado, Boulder, CO, USA.; ^4^ Institute of Physics, University of Amsterdam, 1098 XH Amsterdam, Netherlands.

## Abstract

Driven-dissipative many-body systems are ubiquitous in nature and a fundamental resource for quantum technologies. However, they are also complex and hard to model because they cannot be described by the standard tools in equilibrium statistical mechanics. Probing nonequilibrium critical phenomena in pristine setups can illuminate fresh perspectives on these systems. Here, we use an ensemble of cold ^88^Sr atoms coupled to a driven high-finesse cavity to study the cooperative resonance fluorescence (CRF) model, a classic driven-dissipative model describing coherently driven dipoles superradiantly emitting light. We observe its nonequilibrium phase diagram characterized by a second-order phase transition. Below a critical drive strength, the atoms quickly reach the so-called superradiant steady state featuring a macroscopic dipole moment; above the critical point, the atoms undergo persistent Rabi-like oscillations. At longer times, spontaneous emission transforms the second-order transition into a discontinuous first-order transition. Our observations pave the way for harnessing robust entangled states and exploring boundary time crystals in driven-dissipative systems.

## Teaser

Two non-equilibrium phases, arising from competition between collective emission and a drive, are probed with long-lived dipoles.

## INTRODUCTION

Light-matter interactions lie at the heart of modern quantum technologies. The recent advances in quantum simulation (*1*–*3*), computation (*4*), sensing (*5*), and communication (*6*) rely on the precise control and engineering of atom-light couplings. Another exciting yet challenging frontier is exploring the interaction between light and dense ensembles of radiating dipoles in which the dominant effect is collective emission or superradiance (*7*, *8*). Since the introduction of superradiance, there has been a continuous theoretical effort to understand this effect; superradiance has also been observed in a variety of experimental platforms such as cold atoms (*9*, *10*), matter waves (*11*), waveguides (*12*, *13*), circuit quantum electrodynamics (QED) (*14*), and solid-state systems (*15*, *16*). Furthermore, it has been demonstrated that one can use superradiance for realizing improved optical atomic clocks (*17*, *18*).

Given this progress, a natural next step is to explore what happens when such a system is continuously driven. This scenario has been explored theoretically since the 1970s under the name of cooperative resonance fluorescence (CRF) (*19*–*21*).

This iconic dissipative quantum optics model is known to feature a nonequilibrium second-order phase transition originating from the competition between the coherent drive and collective superradiant emission. This can be compared with open system implementations of the Dicke model (*22*–*29*), which, in contrast, feature an equilibrium phase transition emerging already at the Hamiltonian level, with cavity dissipation modifying the character of the phase transition or leading to the emergence of new dynamical phase transitions. The CRF model is also different from the lasing transition in a continuous superradiant laser (*9*, *30*), in which atoms are incoherently pumped to the excited state.

Despite this interest and the model’s simplicity, experimental effort to demonstrate CRF has been scarce as it proved difficult to see the transition given the fully collective nature of the model and the fast timescales inherent to typical dipole-allowed optical transitions. A recent experiment in free space observed related physics (*31*), although follow-up theoretical studies have found that the spatial propagation of light across the array introduces a notion of locality in the system in the form of anisotropic dipolar interactions. The finite-range nature of these interactions leads to qualitative and quantitative differences between the free space and the all-to-all CRF models and their phases (*32*–*34*).

Here, we report an observation of the second-order superradiant transition in the CRF model by resonantly driving an ensemble of ^88^Sr atoms on a narrow linewidth transition inside of a resonant high-finesse cavity. We identify and distinguish the two predicted phases by observing both spin and light degrees of freedom as the drive strength is varied. Central to our observations are two key elements of the experiment. First, the use of a high-finesse optical cavity allows us to operate with a spatially dilute ensemble of atoms with negligible dipolar or contact interactions; the cavity also provides well-defined phase matching to a single optical mode, ensuring that the atoms are always in a fully collective regime. Second, the interrogation of a narrow-linewidth atomic transition enables us to observe the collective physics of CRF on an experimentally accessible timescale. The clean separation of timescales between the collective decay and single-particle spontaneous decay also allows us to observe how spontaneous emission slowly steers the system toward a qualitatively different steady state that displays a discontinuous transition in both spin and light observables. This physics is related to prior studies in optical bistability (*35*–*39*), although past experimental works have focused mostly on the properties of the light, such as hysteretic transmission, and on timescales where spontaneous emission is important. Our work paves the way for experimental verification of new symmetries in open quantum systems (*40*–*43*) and opens the door for exploring new ways of generating spin squeezing (*43*) and spectroscopy on ultranarrow clock transitions (*44*, *45*).

## RESULTS

### Experimental setup and theoretical model

**Fig. 1.**
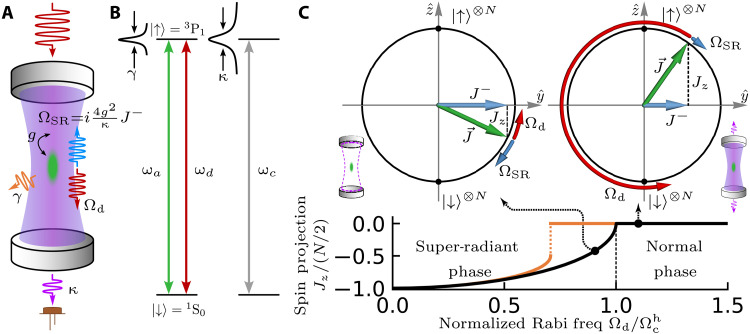
Experimental schematics and the phase diagram of the CRF model. (**A**) ^88^Sr atoms (green) are trapped inside a high-finesse optical cavity (gray). The atom-cavity system is characterized by a RMS single-photon Rabi frequency 2*g*, the excited-state lifetime γ, and a FWHM linewidth of the cavity κ. The injected 689-nm light establishes a drive field (red) with Rabi frequency Ω_d_ inside the cavity. In response, the atoms emit a superradiant field (blue), characterized by Rabi frequency $\Omega_{\text{SR}}=i\frac{4g^{2}}{\kappa }\langle {\hat{J}}^{-}\rangle$. The coherent addition of the two fields (purple) leaks through the cavity mirror. (**B**) Energy-level diagram. The cavity resonance frequency ω_c_ and the drive frequency ω_d_ are both resonant with the S01−3P1(mJ=0) transition ω_a_. (**C**) Phase diagram of the CRF model. The top panels show the Bloch sphere in the *y*-*z* plane and cavity illustrations for the superradiant phase (left) and the normal phase (right), corresponding to the representative points on the phase diagram (bottom row). The external drive Ω_d_ (the superradiant-field Ω_SR_) acts like a torque that tries to rotate the collective Bloch vector $\vec{J}$ counterclockwise (that always tries to bring $\vec{J}$ back to the ground state). In the superradiant phase ($\Omega_{d}<\Omega_{c}^{h}$), the drive and the superradiance balance each other leading to intracavity field *a* = 0, and the atoms reach a steady state with a spin-projection $J_{z}<0$. In the normal phase ($\Omega_{d}>\Omega_{c}^{h}$), the superradiant field is no longer strong enough to cancel the drive, so the Bloch vector starts to Rabi flop, and $J_{z}$ time-averages to zero. The bottom row shows the steady-state $J_{z}$ as a function of normalized Rabi frequency $\Omega_{d}/\Omega_{c}^{h}$, which indicates a second-order phase transition. Spontaneous emission modifies the transition to a first-order one with a smaller critical drive (orange curve).

To explore the phase transition, we trap an ensemble of *N* = 10^3^ to 10^4^ ^88^Sr atoms to interact with a driven high-finesse optical cavity. The atoms are laser-cooled and trapped in the Lamb-Dicke regime by a one-dimensional (1D) optical lattice along the cavity axis at a wavelength of 813 nm. We work along the narrow-linewidth 689-nm transition and treat the ground state ∣↓〉 = ∣^1^S_0_, *m*_*J*_ = 0〉 and the excited state ∣↑〉 = ∣ ^3^P_1_, *m*_*J*_ = 0〉 of each atom as an effective spin-1/2 system with associated spin operators ${\hat{s}}_{i}^{z}=\frac{1}{2}\left(|↑\rangle ,{\langle ↑|}_{i},-,|↓\rangle ,{\langle ↓|}_{i}\right)$ and ${\hat{s}}_{i}^{-}=|↓\rangle \langle ↑{|}_{i}$, where *i* labels the atoms. The excited state has a radiative linewidth of γ = 2π × 7.5 kHz, and the cavity has a full width at half maximum (FWHM) linewidth of κ = 2π × 153 kHz. Atoms are inhomogenously coupled to the standing-wave cavity mode with a spatially averaged root mean square (RMS) single-photon Rabi frequency of 2*g* = 2π × 15 kHz. The RMS single-atom cooperativity is $C=4g^{2}/\kappa \gamma =0.21\left(2\right)$, putting us in the collective strong coupling regime with *NC* ≫ 1 (*46*). In this experiment, we tune the cavity on resonance with the atomic transition and direct a laser beam, also resonant with the atoms, into the cavity to act as a drive (see Fig. 1A).

Under these conditions and assuming for simplicity that all the atoms couple to the cavity identically and with strength *g*, the system can be modeled by the following master equation (Supplementary Text)

$$\begin{array}{c} \frac{d\hat{\rho }}{\mathrm{dt}}=-\frac{i}{\hbar }\left[\hat{H},\hat{\rho }\right]+\kappa \;L_{c}\left(\hat{\rho }\right),\\ \hat{H}/\hbar =g\left({\hat{a}\hat{J}}^{+}+{\hat{a}}^{†}{\hat{J}}^{-}\right)-\frac{\mathrm{i\kappa}}{4g}\Omega_{d}\left(\hat{a}-{\hat{a}}^{†}\right) \end{array}$$ (1)

Here, $\hat{\rho }$ is the density matrix of the full atom-cavity system, ${\hat{J}}^{-}=\sum_{i=1}^{N}{\hat{s}}_{i}^{-}$ and ${\hat{J}}^{+}={\left({\hat{J}}^{-}\right)}^{†}$ are, respectively, collective spin lowering and raising operators that quantify the collective atomic coherence along the two-level system, the operator ${\hat{a}}^{†}$ ($\hat{a}$) creates (destroys) one photon in the cavity mode, and the Lindbladian superoperator $L_{c}\left(\hat{\rho }\right)=\hat{a}\;\hat{\rho }\;{\hat{a}}^{†}-\left\{{\hat{a}}^{†}\hat{a},\hat{\rho }\right\}/2$ describes photon emission through the cavity mirrors. The first term in the Hamiltonian $\hat{H}$ is the Tavis-Cummings atom-cavity interaction, and the second term represents the laser drive with a strength quantified by the Rabi frequency $\Omega_{d}=\alpha \times 2g$, where α is the coherent-state amplitude that would be established inside the cavity by the drive without atoms. For now, we have omitted single-particle spontaneous emission, which happens on a slower timescale set by $\gamma^{-1}$.

We refer to this model as CRF following the nomenclature starting in the 1970s (*21*, *47*). Although we are recasting the model in a broader setting, i.e. including the intracavity field, Eq. 1 gives rise to similar predictions for the steady state [Supplementary Text and (*21*)]. We note that this model has also been referred to both as the driven Dicke model (*31*, *32*, *42*) and as driven superradiance (*48*, *49*).

The properties of the steady state of the system, which satisfies $d{\hat{\rho }}_{\text{ss}}/\mathrm{dt}=0$, can be understood by examining the mean-field equation of motion for the intracavity field $a=\langle \hat{a}\rangle$ and the atomic coherence $J^{-}=\langle {\hat{J}}^{-}\rangle$

$$\begin{array}{c} \frac{\mathrm{da}}{\mathrm{dt}}=\frac{\kappa }{4g}\Omega_{d}-{\mathrm{igJ}}^{-}-\frac{\kappa }{2}a,\\ \frac{{\mathrm{dJ}}^{-}}{\mathrm{dt}}=2{\text{igaJ}}_{z},\frac{{\mathrm{dJ}}_{z}}{\mathrm{dt}}=-\mathrm{ig}\left({\mathrm{aJ}}^{+}-a^{*}J^{-}\right) \end{array}$$ (2)

where $J_{z}=\langle {\hat{J}}_{z}\rangle =\sum_{i}\langle {\hat{s}}_{i}^{z}\rangle$ represents the collective spin projection. In the equation for the field, the first term on the right hand side describes the external drive, the second term describes the superradiant field radiated into the cavity by the collective atomic dipole moment with strength proportional to $J^{-}$, and the last term accounts for the cavity field decay. The other two equations describe how the intracavity field rotates the collective Bloch vector.

There is a unique mean-field stable steady state solution to Eq. 2 characterized by zero intracavity field *a* = 0 and an atomic coherence given by $J^{-}=-i\left(\Omega_{d}/\Omega_{c}^{h}\right)\left(N/2\right)$, where we have defined the critical Rabi frequency $\Omega_{c}^{h}=\mathrm{NC}\gamma /2=2{\mathrm{Ng}}^{2}/\kappa$ for homogeneous couplings. This solution does not exist when $\Omega_{d}>\Omega_{c}^{h}$ since the magnitude of $J^{-}$ must always be smaller than N/2.

The nature of this steady state can be intuitively illustrated using Bloch spheres as shown in Fig. 1C. In our experiment, the spins all start at the south pole $\left({|↓\rangle }^{⊗N}\right)$. The external field rotates the Bloch vector up, causing the transverse coherence $J^{-}$ to increase. This induces the atoms to emit a superradiant field into the cavity, characterized by an effective Rabi frequency $\Omega_{\text{SR}}=i\frac{4g^{2}}{\kappa }J^{-}$, which destructively interferes with the drive. When $\Omega_{d}<\Omega_{c}^{h}$, this field is strong enough to cancel the drive, resulting in a steady state with *a* = 0 and spin projection $J_{z}<0$ that depends on the normalized Rabi frequency $\Omega_{d}/\Omega_{c}^{h}$. We call this the “superradiant phase” because the atoms have a macroscopic dipole moment $\left(|J^{-}|>0\right)$, although the intracavity field is zero. At small drive strength, the zero field in steady state can also be interpreted as a destructive interference between the normal modes in the atom-cavity system (*38*, *50*).

When $\Omega_{d}>\Omega_{c}^{h}$, the superradiant field emitted by the atoms is never large enough to cancel the drive field, leading to a nonzero total electric field inside the cavity. The Bloch vector will just keep rotating, and in the limit where $\Omega_{d}\gg \Omega_{c}^{h}$, the dynamics approach single-particle Rabi oscillation at a frequency $\Omega_{d}$. Hence, we call this the “normal phase.” Phase space diffusion arising from beyond mean-field dissipative effects leads to a steady state with $J_{z}=0$ (*21*) such that at $\Omega_{d}=\Omega_{c}^{h}$, the system exhibits a second-order phase transition, indicated by nonanalytic behavior in $J_{z}$ as shown in Fig. 1C. Because the beyond mean-field steady state in this regime occurs beyond experimentally accessible timescales (*21*, *51*), instead, we identify the normal phase with a measurement of a nonzero cavity field.

### CRF superradiant phase transition

We probe this transition by sending laser light resonant with the atomic dipoles through one of the cavity mirrors. To avoid transient effects, we linearly ramp the Rabi frequency of the drive from 0 to $\Omega_{d}$ over 5 μs. We then hold the laser intensity fixed for 4.3 μs to let the system reach its steady state. To measure the spin projection $J_{z}$ at the end of the hold period, we first shelve the atoms in ∣↑〉 by flashing on a resonant 688-nm laser from the side to optically pump them to metastable states while rapidly turning off the drive and then measure the number of atoms left in ∣↓〉 (see the Materials and Methods section for details). We determine $J_{z}$ at the end of the hold period by comparing this result to the total number of atoms measured before the drive is turned on.

**Fig. 2.**
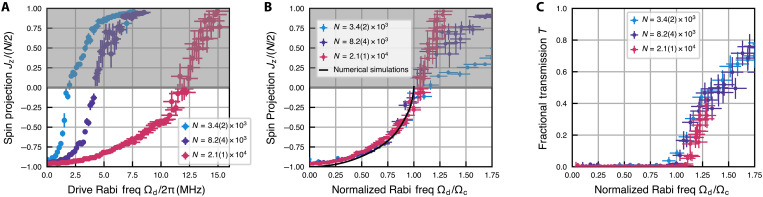
Observing the second-order superradiant phase transition. (**A** and **B**) Steady-state spin projection $J_{z}$ after a 9.3 μs drive as a function of (A) Rabi frequency $\Omega_{d}$ and (B) normalized Rabi frequency $\Omega_{d}/\Omega_{c}$ with critical Rabi frequency $\Omega_{c}=0.35\mathrm{NC}\gamma$. Regions of gray hereafter indicate that our measurement of spin-projection $J_{z}$ does not accurately capture the real $J_{z}$ value of our system at the end of the drive duration (see main text). The three differently colored datasets are for three different total atom numbers *N* with the error bars denoting the standard deviation. In (B), below the critical drive, the three sets at different *N* collapse on top of each other after normalizing to $\Omega_{c}$, which at the same time align well with the theory prediction (solid black curve). (**C**) Transmission *T* of the cavity with atoms inside normalized to that of a cavity without atoms as a function of normalized Rabi frequency $\Omega_{d}/\Omega_{c}$. The transmitted power is averaged over the last 5 μs of the drive duration. The measured transmission is consistent with 0 below the critical Rabi drive $\Omega_{c}$, which indicates the complete cancellation of the external drive field by the superradiantly emitted field; the fact that transmission becomes finite above the critical drive $\Omega_{c}$ confirms that the superradiant field is no longer large enough to cancel the drive in the normal phase. Error bars in the plots and hereafter represent the SEM unless otherwise noted.

**Fig. 3.**
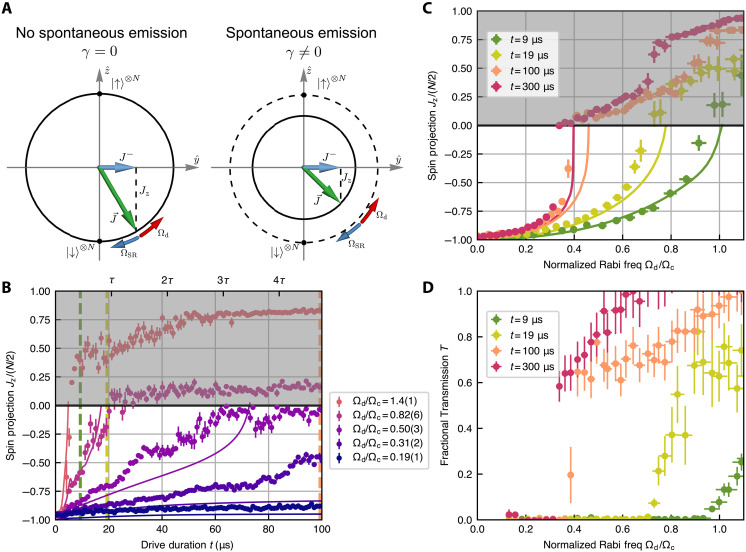
Modification of critical behavior by single-particle spontaneous emission. (**A**) Bloch sphere illustration of the superradiant phase steady state without (left) and with (right) spontaneous emission. On the left, the steady-state Bloch vector $\vec{J}$ stays in the collective manifold, and the torque coming from the external drive $\Omega_{d}$ and superradiant field $\Omega_{\text{SR}}$ balance. On the right, $\vec{J}$ is no longer in the collective manifold. $\vec{J}$ is rotated up from the south pole to increase the coherence $J^{-}$ such that the superradiant field can still cancel the external drive. Consequently, the steady-state $J_{z}$ on the right is always higher than that on the left. (**B**) $J_{z}$ as a function of drive duration *t*. Different colors represent different drive strengths with fixed atom number. The solid lines represent the results of numerical simulation. The tick marks on the top horizontal axis indicate the drive duration as a function of $\tau =\gamma^{-1}=21$ μs. Traces with $\Omega_{d}/\Omega_{c}=0.50,0.82$ have $J_{z}$ slowly relax above 0 on a timescale set by τ, while the traces with $\Omega_{d}/\Omega_{c}=0.19,0.31$ have $J_{z}$ remain below 0 and stay in the superradiant phase. The colored vertical dashed lines indicate the drive durations corresponding to the data with matching colors in (C) and (D). (**C**) $J_{z}$ as a function of $\Omega_{d}/\Omega_{c}$ with fixed atom number. Different colors indicate different drive durations *t* before the measurement of $J_{z}$. The critical point, where $J_{z}$ crosses 0, reduces to smaller drive values as the drive duration increases, and the continuous second-order phase transition (*t* = 9 μs, green curve) turns into a first-order transition (*t* = 300 μs, red curve), where a clear jump is observed. (**D**) Fractional transmission *T* as a function of $\Omega_{d}/\Omega_{c}$. The green curve displays a smooth transition, while the red curve with a longer drive duration displays a discontinuity at the critical point.

**Fig. 4.**
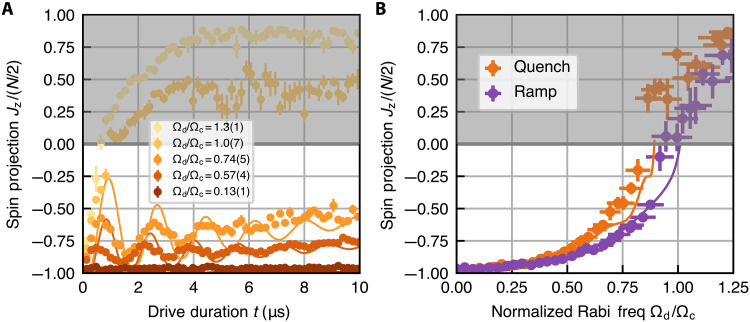
Quench response of driven emitters in the resolved VRS regime. (**A**) Spin-projection $J_{z}$ as a function of the total drive duration *t* accompanied by theory predictions (solid curves). The data are taken by suddenly turning on the drive in 100 ns and holding the drive constant for a variable amount of time before extinguishing the drive and measuring the spin projection $J_{z}$. Traces with different colors represent different drive strengths with fixed atom number $N=8.0\left(8\right)\times 10^{3}$. The traces with $\Omega_{d}/\Omega_{c}=0.57,0.74$ exhibit characteristic oscillations associated with the vacuum Rabi frequency $2g\sqrt{N}$. The trace with $\Omega_{d}/\Omega_{c}=1.0$ should normally relax to $J_{z}$ = 0 as it is at the critical point of the CRF superradiant transition, but the quench causes $J_{z}$ quickly to shoot above 0 in 1 μs, after which it remains in the normal phase. (**B**) Spin-projection $J_{z}$ as a function of $\Omega_{d}/\Omega_{c}$ at the end of a 9.3 μs drive for quenching (orange) and ramping on the drive (purple) with $N=8.3\left(3\right)\times 10^{3}$. The ramping procedure is the same as that for Fig. 2. Both datasets agree well with numerical simulations (solid curves). However, for the same $\Omega_{d}$, the measured $J_{z}$ in quenched experiments is consistently above the $J_{z}$ obtained in ramped experiments. This suggests that with a quench, the system can overshoot and fail to relax to the true steady state predicted by the mean-field theory (purple solid line) on experimental timescales.

**Fig. 5.**
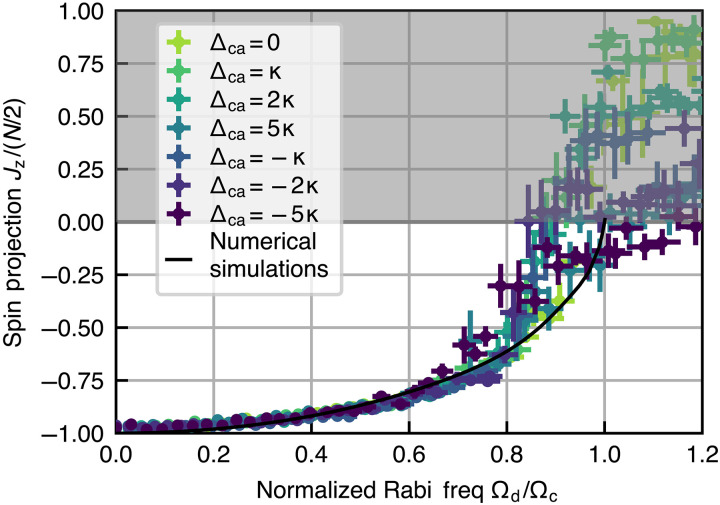
Invariance of the critical point to the detuning $\Delta_{\text{ca}}$ of the cavity from atomic resonance. We show the measured spin projection $J_{z}$ as a function of $\Omega_{d}$, defined as the Rabi frequency that would be established in the bare cavity when $\Delta_{\text{ca}}=0$, normalized to the critical Rabi frequency $\Omega_{c}=0.35\mathrm{NC}\gamma$. The different colors of data are taken at different cavity-atom detunings $\Delta_{\text{ca}}$ spanning many bare cavity linewidths (from −5κ to +5κ) and with fixed atom number, while the drive is kept on resonance with the atoms ($\omega_{d}=\omega_{a}$). Despite such large changes, the datasets all overlap with each other below the transition, showing the predicted insensitivity to the cavity detuning. Full numerical simulations suggest that the dependence on detuning on $\Delta_{\text{ca}}$ is small despite the presence of inhomogeneous coupling (Supplementary Text); so for clarity here, we only show simulations for $\Delta_{\text{ca}}=0$ (black curve).

We show the (normalized) spin projection $J_{z}/\left(N/2\right)$ as a function of Rabi frequency $\Omega_{d}$ for three different atom numbers in Fig. 2A. After normalizing the Rabi frequency by $\Omega_{c}=0.70\Omega_{c}^{h}=0.35\mathrm{NC}\gamma$, we find that the curves in the region $\Omega_{d}/\Omega_{c}<1$ collapse on top of each other, certifying the collective nature of the steady state. The numerical factor 0.70 mainly accounts for inhomogenous atom-cavity coupling, which reduces the critical Rabi drive but does not change the second-order nature of the transition (Supplementary Text). The collapsed data is in good agreement with numerical simulations (solid black curve), consistent with the prediction of the superradiant phase. In the region where $\Omega_{d}>\Omega_{c}$, our shelving procedure does not properly capture the spin projection $J_{z}$ at the end of the hold period. This is because in this regime, the cavity is populated with a macroscopic field that rapidly transfers atoms from ∣↓〉 to ∣↑〉 where they are then subsequently shelved, leading to a misinferred value of $J_{z}>0$. To emphasize this, we shade the regions with $J_{z}>0$ in gray in Figs. 2 to 5. Note, however, that for smaller atom number, we expect the intracavity field to be relatively small just above the transition, allowing the shelving method to provide a more accurate inference of $J_{z}$. We observe in Fig. 2B that for $N=3.4\times 10^{3}$, the measured value of $J_{z}$ exhibits an abrupt change of behavior at $\Omega_{d}=\Omega_{c}$.

To verify the presence of a phase transition at $\Omega_{d}=\Omega_{c}$, we also directly probe the light degree of freedom. We infer the presence or absence of an intracavity field by measuring the transmitted power normalized to that of an empty cavity, averaged over the last 5 μs of the drive duration, which we call the fractional transmission *T*. This quantity establishes the extent to which the superradiant field is able to cancel the external drive. We measure *T* while varying the drive strength $\Omega_{d}$ between different experimental shots and plot the results in Fig. 2C. For $\Omega_{d}<\Omega_{c}$, we observe a value consistent with zero transmitted power, in accordance with the theoretical expectations. For $\Omega_{d}>\Omega_{c}$, the transmitted power rises very sharply, demonstrating that the superradiant field can no longer cancel the drive in the normal phase. The value of the drive separating these two regions coincides with the point where the spin projection $J_{z}$ approaches 0 from below in our atomic observable. These two pieces of evidence together allow us to identify $\Omega_{c}$ as a critical point which separates the superradiant phase and the normal phase.

### Melting into a first-order transition

By driving the system for longer times, we can explore the effect of a different type of dissipation: spontaneous emission, which is a noncollective effect described by another Lindblad term added to the master equation Eq. 1, $\gamma L_{\text{se}}\left(\hat{\rho }\right)=\gamma \sum_{i}\left({\hat{s}}_{i}^{-}\hat{\rho }{\hat{s}}_{i}^{+}-\left\{{\hat{s}}_{i}^{+}{\hat{s}}_{i}^{-},\hat{\rho }\right\}/2\right)$. Unlike superradiant decay, which preserves the total length of the Bloch vector $J\approx \sqrt{{|J^{-}|}^{2}+J_{z}^{2}}$, spontaneous emission shortens J. If the system starts in the steady state of the superradiant phase in the CRF model, then the atomic coherence $J^{-}$ is shortened by spontaneous emission and in turn radiates a smaller field. This leads to a nonzero net field inside the cavity due to the imbalance of the drive and superradiant field. The Bloch vector then rotates upward, which restores $J^{-}$ to its previous value but introduces an effective upward force on $J_{z}$ that scales with the drive strength $\Omega_{d}$ (Supplementary Text). At the same time, the decay to the ground state induced by spontaneous emission leads to a competing process that pushes $J_{z}$ down toward −N/2. This competition leads to two different behaviors depending on the drive strength. For strong drive strengths, the upward force causes $J_{z}$ to slowly relax toward 0 while holding $J^{-}$ constant. Once $J_{z}$ reaches 0, the atoms start to undergo Rabi-like oscillation, and the cavity gets quickly populated by photons, indicative of the normal phase despite having started in the CRF superradiant phase. However, when $\Omega_{d}$ is small enough, since the upward force on $J_{z}$ scales with $\Omega_{d}$, $J_{z}$ will not relax all the way toward 0. Instead, the Bloch vector arrives at a new steady state where the upward and downward forces balance, and the superradiant and drive fields retain a near-perfect cancellation despite the reduction in spin length *J* (Supplementary Text). This corresponds to the superradiant phase (see Fig. 3A), which is analogous to the “cooperative branch” in the bistability literature (Supplementary Text) (*35*, *52*). These two regimes are separated by a new critical point $\Omega_{c}^{\prime }$, and the spin projection $J_{z}$ will now display a discontinuous jump at this point as opposed to the continuous transition in the CRF model (*21*, *53*) (also see Fig. 1C).

We show traces of the spin projection $J_{z}$ as a function of the drive duration t for different drive strengths in Fig. 3B. At low drives $\left(\Omega_{d}/\Omega_{c}=0.19,0.31\right)$, $J_{z}$ reaches its fully collective superradiant steady state at short times, and at longer times, the Bloch vector still remains below the south pole with a modified value. As the drive strength is increased $\left(\Omega_{d}/\Omega_{c}=0.50,0.82\right)$, the spin projection $J_{z}$ remains below the equator on a timescale shorter than the excited state lifetime $\tau =\gamma^{-1}=21$ μs. For longer times, however, it slowly moves toward 0, consistent with theoretical expectations. At even larger drives ($\Omega_{d}/\Omega_{c}=1.4$), the inversion quickly rises above 0, suggesting that the system is in the normal phase both at short and long times.

We characterize the gradual change in $J_{z}$ over time by providing snapshots of $J_{z}$ as a function of the Rabi frequency $\Omega_{d}$ for different drive durations in Fig. 3C. At short times (9 μs), we observe a continuous superradiant phase transition, consistent with Fig. 2B. At longer times (t≳τ), spontaneous emission becomes important and noticeably shifts the transition point, characterized by the point where $J_{z}$ crosses 0, to a lower value of $\Omega_{d}$. By t=100 μs, the system has already equilibrated to a new steady state, and the spin projection displays a discontinuous jump at $\Omega_{d}/\Omega_{c}=0.4$. These observations can be correlated with the results of fractional transmission, *T*, through the cavity, shown in Fig. 3D as a function of $\Omega_{d}$ for different drive durations. Again, the transition points where the transmission rises above 0 coincide with the points at which $J_{z}$ reaches 0 in Fig. 3C.

### Short-time dynamical response

So far, we have focused on the steady state and long-time behavior of $J_{z}$ in our system, which follows the predictions of the original CRF model (*21*). Nevertheless, our cavity system operates in a very different regime, characterized by a resolved vacuum Rabi splitting (VRS) $2g\sqrt{N}>\kappa$ (“resolved VRS regime” hereafter). Under these conditions, excitations can be coherently exchanged between the atoms and the photon field, and the Bloch vector behaves similar to an underdamped oscillator (*54*). In contrast, the original CRF Lindbladian arises in the “nonresolved VRS” regime ($2g\sqrt{N}\ll \kappa$) in which the cavity field adiabatically follows the atoms [(*10*) and Supplementary Text], corresponding to an overdamped oscillator. We explore this distinction by measuring the dynamical response of our system when we suddenly turn on the drive $\Omega_{d}$ rather than slowly ramping the amplitude of the drive as we did in Fig. 2.

In this experiment, we quench the drive strength from 0 to $\Omega_{d}$ in approximately 100 ns and then hold for a total drive duration *t*. After the quench, the atoms and the intracavity field will exhibit transient behavior as they settle to the steady state. To illustrate this, we show time traces of the spin projection $J_{z}$ as a function of the drive duration in Fig. 4A. At low drive strengths, we observe oscillations in $J_{z}$ at a frequency consistent with $g\sqrt{N}$, with $N=8.0\left(8\right)\times 10^{3}$, characteristic of dynamics in the resolved VRS regime. The frequency of those oscillations aligns well with theoretical predictions, although the amplitude is observed to be smaller due to additional dephasing mechanisms in the experiment not accounted for in theory such as atomic motion (*55*, *56*).

While the ramp used in Fig. 2 is designed to be slow enough to prepare the system close to the superradiant steady state, if the system is far away from this steady state and then is suddenly subjected to a quenched drive, then the atoms will not have enough time to generate a field to cancel the external drive and may start to oscillate. Hence, the resolved VRS regime features an additional region of bistability compared to the nonresolved VRS regime (*57*) where the short-time dynamical behavior of the system, either settling into the CRF steady state or oscillating persistently, is determined by the initial conditions. We can characterize this effect by comparing the $J_{z}$ values obtained after driving the system for 9.3 μs following a quench or ramp of the drive. Figure 4B shows, consistently with theory predictions, that the post-quench $J_{z}$ is always above the post-ramp $J_{z}$, and the difference increases as we approach the critical point $\Omega_{d}/\Omega_{c}=1$. Moreover, for $\Omega_{d}/\Omega_{c}$ slightly smaller than 1, we observe that the system appears to enter the normal phase after a quench, although it remains in the superradiant phase after a ramp. This highlights how, in the resolved VRS regime, our ability to access the superradiant phase can be influenced by the dynamical response to the drive.

### Invariance of the critical point under different cavity detunings

In the nonresolved VRS limit, in which the cavity field can be adiabatically eliminated, detuning the cavity from resonance by $\Delta_{\text{ca}}=\omega_{c}-\omega_{a}$ gives rise to elastic spin exchange interactions $\chi {\hat{J}}^{+}{\hat{J}}^{-}$, with the ratio of elastic interactions to inelastic collective decay (with rate Γ) determined by $\chi /\Gamma =\Delta_{\text{ca}}/\kappa$ (*58*). Based on previous work in this limit (*51*), the elastic and inelastic interactions combine to keep the critical point separating the superradiant and normal phase independent of $\Delta_{\text{ca}}$ as long as the drive remains on resonance with the atomic transition (Supplementary Text).

The invariance of the critical point to cavity detuning applies more generally, holding even in the resolved VRS limit studied in this work. At a high level, this can be understood by treating the cavity mode as a harmonic oscillator. Both the externally applied laser and the atomic dipole $J^{-}$ drive this oscillator with the same detuning $\Delta_{\text{ca}}$ such that the steady-state field that builds up in response to these two drives is modified in a common-mode way and can therefore still cancel. Formally, the steady-state field established inside the bare cavity by the external drive is described by a modified Rabi frequency $\Omega_{d}\left(\Delta_{\text{ca}}\right)=\Omega_{d}/\left(1+i\frac{\Delta_{\text{ca}}}{\kappa /2}\right)$, where $\Omega_{d}$ is, as defined before, the Rabi frequency that would be established in the cavity when $\Delta_{\text{ca}}=0$. Correspondingly, the field established by the radiating dipole without an external drive would have a Rabi frequency $\Omega_{\text{SR}}\left(\Delta_{\text{ca}}\right)=i\frac{4g^{2}}{\kappa }J^{-}\left(\Delta_{\text{ca}}\right)/\left(1+i\frac{\Delta_{\text{ca}}}{\kappa /2}\right)$. Equating these two Rabi frequencies yields a steady-state solution $J^{-}\left(\Delta_{\text{ca}}\right)=-i\left(\Omega_{d}/\Omega_{c}^{h}\right)\left(N/2\right)$, which is independent of $\Delta_{\text{ca}}$, suggesting that the steady-state $J_{z}$ has no detuning dependence. Notably, the relative phase between the steady-state $J^{-}$ and the external applied drive $\Omega_{d}$ also does not change, even in the presence of a cavity detuning.

To validate these expectations, we ramp and hold the drive for 9.3 μs and then measure the spin projection $J_{z}$ versus drive strength for different atom-cavity detunings $\Delta_{\text{ca}}$. As shown in Fig. 5, we indeed see that, below the critical point, the different traces behave similarly, showing that the steady state $J_{z}$ does not depend strongly on the cavity detuning $\Delta_{\text{ca}}$. We highlight that when varying the cavity detuning over the range ±5κ, the intracavity field established by the drive in the absence of atoms (or equivalently $|\Omega_{d}\left(\Delta_{\text{ca}}\right)|$) is reduced by a factor of 10, and therefore, the fact that the steady state does not significantly change demonstrates its insensitivity to the cavity detuning over a wide parameter regime.

## DISCUSSION

In summary, we have observed the superradiant phase transition in the CRF model predicted more than 40 years ago. We also witnessed how spontaneous emission melted the continuous transition into a first-order transition. In the future, it will be interesting to explore the properties of the steady states in the presence of additional Hamiltonian interactions (*42*) or at the level of quantum fluctuations because the spins get more squeezed as the drive strength approaches the critical point from below (*21*, *59*). Alternatively, by introducing a third atomic level to our system, one can also potentially generate squeezed states between the ground state and the third state via the generation of a dissipative Berry phase (*43*). These schemes are all compatible with ultralong-lived clock transitions in alkaline earth atoms and thus can be used to improve state-of-the-art atomic clocks (*60*, *61*). Furthermore, carefully monitoring the light leaking out of the cavity, in combination with post-selection and feedback operations, opens up the possibility of implementing exotic out-of-equilibrium phenomena such as measurement-induced phase transitions (*62*, *63*).

## MATERIALS AND METHODS

### Experimental procedure

Each shot of the experiment starts by loading *N* = 10^3^ to 10^4^ ^88^Sr atoms into a 1D 813-nm optical lattice inside a high-finesse optical cavity. The thermal cloud has a temperature of about 15 μK. After loading into the lattice as shown in fig. S1A, we apply a magnetic field of $\vec{B}=1.5\;G\;\hat{x}$. The lattice is linearly polarized in the *xz* plane, and its polarization is adjusted to 52° with respect to $\vec{B}$ to reduce the differential ac stark shift on the ∣^1^S_0_〉 − ∣^3^P_1_, *m*_J_ = 0〉 transition (the π transition) (*56*, *64*). We observe a residual (lattice-induced) maximum differential ac shift of 125(25) kHz between the ground ∣↓〉 and the excited state ∣↑〉.

We then measure the initial total atom number via the magnitude of the VRS. This is accomplished by ramping the frequency of an $\vec{x}$-polarized probe over 5 MHz in 10 ms and recording the transmitted power on a single-photon counting module (*46*, *65*) (see fig. S1).

We begin the actual experiment by coherently driving the cavity with $\vec{x}$-polarized light resonant with the π transition. The drive incident on the cavity has a temporal profile as shown in fig. S1B. For the experiment where we ramp the drive, we linearly ramp the incident field $E\left(\tilde{t}\right)$ over time $\tilde{t}$ to the desired maximum incident field *E*, such that this maximum field would establish a Rabi frequency of $\Omega_{d}$ inside a bare cavity in steady state, and then hold the field strength fixed. We refer to the total length of the drive and hold operation as the drive duration *t*.

To track the intracavity field, we monitor the cavity transmission using heterodyne detection. For each drive strength, we separately measure the transmitted power with no atoms in the cavity to obtain the fractional (power) transmission *T*.

To measure the spin projection $J_{z}$ at the end of the drive duration, we both turn off the incident drive and rapidly freeze the dynamics by flashing on a 688-nm beam resonant with the excited state ${\;}^{3}P_{1}-{\;}^{3}S_{1}$ transition. This optically pumps or shelves the excited state atoms into the metastable states ^3^P_0_ and ^3^P_2_, where they do not interact with the cavity (see next section for details). We can then count the number of atoms left in the ground state via another measurement of the VRS as described previously. Combined with knowledge of the total atom number, we can then infer $J_{z}$ at the end of the drive duration.

For the data in Fig. 5 where the cavity-atom detuning satisfies $\Delta_{\text{ca}}\neq 0$, we account for the dispersive shift when extracting atom numbers from the normal mode splitting acquired before and after the actual experiment. For each data point of the spin projection $J_{z}$ and fractional transmission *T* presented in the main text, we average over at least 8 shots of the experiment.

### Shelving protocol

We shelve the atoms that are in ∣^3^P_1_, *m*_J_ = 0〉 at the end of the drive duration by shining 688-nm laser resonant with the ^3^P_1_ − ^3^S_1_ transition that radiatively decays into the metastable state at a rate γ_shelve_/2π = 8.1 MHz. To ensure a high on/off extinction ratio of the 688-nm beam and a rapid switching time, we send the 688-nm beam first through an acoustic-optic modulator (AOM) and then a fiber electro-optic modulator (EOM) to control the amplitude and frequency of the 688-nm laser. The frequency shifting diagram of the 688-nm beam is shown in fig. S2B. We turn on the 688-nm AOM 50 ns before we turn off the 689 resonant drive. The 688-nm AOM has a rise time of 40 ns. As the 689-nm drive is turned off via switching off the radio frequency signal to the AOM, we send a 5-GHz tone to the fiber EOM, placing a sideband of 688-nm light on resonance with ∣^3^S_1_〉 and obtaining a shelving beam with a Rabi frequency of 25 MHz. The whole shelving procedure lasts for 1 μs.

### Model and simulations

We model the experimental system using a modified version of the master equation given in Eq. 1

$$\frac{d\hat{\rho }}{\mathrm{dt}}=-\frac{i}{\hbar }\left[{\hat{H}}_{\text{tot}},\hat{\rho }\right]+\kappa \;L_{c}\left(\hat{\rho }\right)+\gamma \;L_{\text{se}}\left(\hat{\rho }\right)$$ (3)

where the Hamiltonian can be expressed as the sum of three pieces ${\hat{H}}_{\text{tot}}={\hat{H}}_{a}+{\hat{H}}_{c}+{\hat{H}}_{\text{int}}$, defined by

$$\begin{array}{c} {\hat{H}}_{a}=\sum_{k=1}^{N}\hbar \left(\omega_{a}+\delta_{k}\right){\hat{s}}_{k}^{z}\\ {\hat{H}}_{c}=\hbar \omega_{c}{\hat{a}}^{†}\hat{a}-i\hbar \sqrt{\kappa }\alpha_{\text{in}}\left(\hat{a}e^{i\omega_{d}t}-{\hat{a}}^{†}e^{-i\omega_{d}t}\right)\\ {\hat{H}}_{\text{int}}=\sum_{k=1}^{N}\hbar g_{k}\left({\hat{a}\;\hat{s}}_{k}^{+}+{\hat{a}}^{†}{\hat{s}}_{k}^{-}\right) \end{array}$$ (4)

which account for the atoms, the driven cavity, and the atom-light interactions, respectively. Here, $\omega_{a}$ is the transition frequency between the two atomic states, $\omega_{c}$ is the cavity resonance frequency, and $\omega_{d}$ is the frequency of the driving laser, which carries $\alpha_{\text{in}}^{2}$ photons/s where we have chosen $\alpha_{\text{in}}$ to be real. The spatial dependence of the cavity electric field gives rise to inhomogeneity in the couplings, given by $g_{k}=g_{0}\text{cos}\left(k\;\delta \phi \right)$, where, $\delta \phi =\pi \lambda_{l}/\lambda_{c}$ and $2g_{0}$ is the single-photon Rabi frequency at an antinode of the cavity mode. This inhomogeneity is caused by the incommensurability of the lattice ($\lambda_{l}$ = 813 nm) and cavity mode ($\lambda_{c}$ = 689 nm) wavelengths. In reality, each coupling $g_{k}$ is associated with a lattice site instead of an atom, but the large number of lattice sites (≈10^3^) makes this distinction unnecessary. For definiteness, we will express all of the couplings in terms of the RMS coupling $g\equiv g_{\text{RMS}}=g_{0}/\sqrt{2}$. This is the convention followed in the main text. We also include inhomogeneous broadening of the atomic transition, accounted for by the $\delta_{k}$ terms, which is caused by small ac Stark shifts due to the trapping lattice.

Dissipation is modeled by the Lindblad superoperators

$$\begin{array}{c} \kappa \;L_{c}\left(\hat{\rho }\right)=\frac{\kappa }{2}\left(2\hat{a}\hat{\rho }\;{\hat{a}}^{†}-{\hat{a}}^{†}\hat{a}\;\hat{\rho }-\hat{\rho }\;{\hat{a}}^{†}\hat{a}\right)\\ \gamma \;L_{\text{se}}\left(\hat{\rho }\right)=\frac{\gamma }{2}\sum_{k=1}^{N}\left(2{\hat{s}}_{k}^{-}\;\hat{\rho }\;{\hat{s}}_{k}^{+}-{\hat{s}}_{k}^{+}\;{\hat{s}}_{k}^{-}\;\hat{\rho }-\hat{\rho }\;{\hat{s}}_{k}^{+}\;{\hat{s}}_{k}^{-}\right) \end{array}$$ (5)

which account for photon leakage through the cavity mirrors with rate κ=2π×153 kHz and spontaneous emission of photons into free space with rate γ=2π×7.5 kHz.

In the rotating frame of the drive, assuming that $\omega_{d}=\omega_{a}$ (atom-drive resonance) and that there is no broadening ($\delta_{k}$ = 0), the Hamiltonian is given by

$${\hat{H}}_{\text{tot}}^{\prime }/\hbar =\Delta_{\text{ca}}{\hat{a}}^{†}\hat{a}+\sum_{k=1}^{N}g_{k}\left({\hat{a}\;\hat{s}}_{k}^{+}+{\hat{a}}^{†}{\hat{s}}_{k}^{-}\right)-i\sqrt{\kappa }\alpha_{\text{in}}\left(\hat{a}-{\hat{a}}^{†}\right)$$ (6)

where $\Delta_{\text{ca}}=\omega_{c}-\omega_{a}$ is the atom-cavity detuning. We recover Eq. 1 by setting $\Delta_{\text{ca}}=0$, omitting γ, and replacing $g_{k}\to g$. In the absence of atoms, these equations lead to a steady-state cavity field $\langle \hat{a}\rangle =2\alpha_{\text{in}}/\sqrt{\kappa }$, which establishes an intracavity Rabi frequency (for the RMS coupler) equal to $\Omega_{d}=4g\alpha_{\text{in}}/\sqrt{\kappa }$. In the main text, we express the strength of the laser drive in terms of $\Omega_{d}$ instead of $\alpha_{\text{in}}$.

Numerical simulations are done by solving Eq. 3 in the mean field approximation. More concretely, we calculate the exact equations of motion for $\langle {\hat{s}}_{k}^{\;z}\rangle$, $\langle {\hat{s}}_{k}^{-}\rangle$, and $\langle \hat{a}\rangle$ and factorize the operator product $\langle {\hat{a}\;\hat{s}}_{k}^{\;z}\rangle \to \langle \hat{a}\rangle \langle {\hat{s}}_{k}^{\;z}\rangle$. To reduce the computational complexity of the simulation, we group the *N* ≈ 10^4^ atoms into $N_{\text{eff}}$ groups of $N/N_{\text{eff}}$ particles with the k′ ensemble of $N_{\text{eff}}$ atoms having a cavity coupling $g_{k\prime }=g_{0}\text{cos}\left[2\pi k\prime /N_{\text{eff}}\right]$ and $k\prime =1,\;\ldots N_{\text{eff}}$. By varying $N_{\text{eff}}$, we find empirically that for $N_{\text{eff}}=30$, the simulations in the superradiant phase have converged, and there is no further need to increase $N_{\text{eff}}$.

The presence of inhomogeneous coupling means that the result of the measurement of atom numbers, and hence, $J_{z}$ is a weighted average of the individual atomic inversions

$$J_{z}/N\to \frac{1}{\sum_{k}g_{k}^{2}}\sum_{k}g_{k}^{2}\langle {\hat{s}}_{k}^{\;z}\rangle$$ (7)

instead of the total inversion. This weighted inversion is also −1 when all the atoms are in the ground state (note that $\langle {\hat{s}}_{k}^{\;z}\rangle =-1/2$ when atom *k* is in the ground state).

As shown in the Supplementary Materials, the modifications introduced by inhomogeneity in the couplings ($g_{k}$) and by the broadening of the atomic transition ($\delta_{k}$, with RMS variation of the same size as γ in our system) do not change the qualitative nature of the second-order superradiant transition nor of the first-order transition at longer times, but they do introduce a renormalization of the critical drives, as accounted for in the main text.

## Supplementary Materials

### This PDF file includes:

Supplementary Text Figs. S1 to S6 Table S1 References
