# Supplementary material for: A dissipation-induced superradiant transition in a strontium cavity-QED system
Source: Sci Adv. 2025 Apr 25;11(17):eadu5799. doi: 10.1126/sciadv.adu5799 (PMC12024687; doi:10.1126/sciadv.adu5799)
Supplement: Supplementary file 1 — Supplementary Text Figs. S1 to S6 Table S1 References [file sciadv.adu5799_sm.pdf]

Science Advances
AAAS

Supplementary Materials for

A dissipation-induced superradiant transition in a strontium cavity-QED system

Eric Yilun Song *et al.*

Corresponding author: Eric Yilun Song, ericyilunsong@gmail.com

Sci. Adv. **11**, eadu5799 (2025)
DOI: 10.1126/sciadv.adu5799

This PDF file includes:

Supplementary Text
Figs. S1 to S6
Table S1
References

Supplementary Text

In this Supplementary Information we (1) describe the relation between our experiment and the original models of cooperative resonance fluorescence, (2) write down the theoretical model describing our experiment including all relevant technical imperfections and (3) provide theoretical analysis, at various degrees of theoretical complexity, of the steady state/dynamical behaviour of the system in the regimes explored in our experiment.

Relation to cooperative resonance fluorescence

In this section, we discuss the relation between our experimental system and the original models of cooperative resonance fluorescence (*21*). Our experimental system is described by the following master equation

$$\dot{\hat{\rho}} = -i\left[g\left(\hat{a}^\dagger\hat{J}^- + \hat{a}\hat{J}^+\right) - \frac{i\kappa\Omega_d}{4g}(\hat{a}-\hat{a}^\dagger), \hat{\rho}\right] + \kappa\left(\hat{a}\hat{\rho}\hat{a}^\dagger - \frac{\{\hat{a}^\dagger\hat{a}, \hat{\rho}\}}{2}\right), \tag{S1}$$

where $\hat{a}$ ($\hat{a}^\dagger$) are annihilation (creation) operators and $\hat{J}^{\pm,z}$ are collective spin $N/2$ operators. Eq. (S1) has three ingredients: the Tavis-Cummings interaction ($\propto 2g$, the single-photon Rabi frequency), the laser pump through the cavity mirror ($\propto \Omega_d$) and loss of photons through the cavity mirror ($\propto \kappa$, the cavity power linewidth). Cooperative resonance fluorescence is described by a different master equation

$$\dot{\hat{\rho}} = -\frac{i\Omega_d}{2}\left[\hat{J}^+ + \hat{J}^-, \hat{\rho}\right] + \Gamma\left(\hat{J}^-\hat{\rho}\hat{J}^+ - \frac{\{\hat{J}^+\hat{J}^-, \hat{\rho}\}}{2}\right), \tag{S2}$$

which involves only the atomic degrees of freedom and describes the competition between a Rabi drive ($\propto \Omega_d$) and superradiant decay ($\propto \Gamma$).

Eq. (S1) reduces to Eq. (S2) (with $\Gamma = 4g^2/\kappa$) when $2g\sqrt{N} \ll \kappa$, but it turns out that for low drives (superradiant phase) and N large, the atomic steady states of both equations are identical and independent of the relation between κ and $g\sqrt{N}$. For larger drives (normal phase) both Eq. (S1) and Eq. (S2) predict persistent oscillations but the details do depend on the ratio between $g\sqrt{N}$ and κ. In the following subsections we include

1. A schematic derivation of Eq. (S2) from Eq. (S1) when $g\sqrt{N} \ll \kappa$ (this is not the regime we operate in, but we include this for completeness)

2. The analytical solution of the steady state Eq. (S1) in the superradiant phase ($\Omega_d < 2g^2N/\kappa$) for N large

3. A discussion of the normal [Rabi flopping, ($\Omega_d > 2g^2N/\kappa$)] phase in both models

Adiabatic elimination of the cavity mode We include this derivation for completeness, although it is valid only when $g\sqrt{N} \ll \kappa$, which is not the regime we operate in [in our experiment $g\sqrt{N} \sim (7-10)\kappa$]. As discussed in the main text, in the absence of the atoms the laser pump would establish an intracavity field of size $\Omega_d/2g$. The connection to cooperative resonance fluorescence can be made manifest by defining the operator $\hat{b}$

$$\hat{b} = \hat{a} - \frac{\Omega_d}{2g}, \tag{S3}$$

which measures the field with respect to $\Omega_d/2g$. The master equation becomes

$$\dot{\hat{\rho}} = -i\left[g\left(\hat{b}^\dagger\hat{J}^- + \hat{b}\hat{J}^+\right) + \frac{\Omega_d}{2}(\hat{J}^+ + \hat{J}^-), \hat{\rho}\right] + \kappa\left(\hat{b}\hat{\rho}\hat{b}^\dagger - \frac{\{\hat{b}^\dagger\hat{b}, \hat{\rho}\}}{2}\right). \tag{S4}$$

In these variables, the Rabi drive acting on the atoms is now explicit. If κ is large compared with the typical timescales of the atomic dynamics, the cavity field $\hat{b}$ will follow adiabatically the atoms. This can be seen from the equation of motion for $\langle\hat{b}\rangle$:

$$\frac{d\langle\hat{b}\rangle}{dt} = -\frac{\kappa}{2}\langle\hat{b}\rangle - ig\langle\hat{J}^-\rangle. \tag{S5}$$

For κ large we can neglect the time derivative and are led to the schematic equivalence $\hat{b} \to -2ig\hat{J}^-/\kappa$. Performing this replacement in Eq. (S4) leads to (see Ref. (*66*) for a rigorous derivation)

$$\dot{\hat{\rho}} = -i\left[\frac{\Omega_d}{2}(\hat{J}^+ + \hat{J}^-), \hat{\rho}\right] + \frac{4g^2}{\kappa}\left(\hat{J}^-\hat{\rho}\hat{J}^+ - \frac{\{\hat{J}^+\hat{J}^-, \hat{\rho}\}}{2}\right), \tag{S6}$$

which is Eq. (S2). The evolution described by Eq. (S6) occurs on timescales Ω_d, $4g^2N/\kappa$, so self-consistency requires both $\Omega_d \ll \kappa$ and $g\sqrt{N} \ll \kappa$.

Steady state in superradiant phase In this subsection, we show that the steady states of Eq. (S1) and Eq. (S2) are identical below the transition point when N is large independent of the relative sizes of $g\sqrt{N}$ and κ. When N is large, mean field theory may provide an accurate description of the steady state, though this assumption needs to be checked by computing fluctuations about

the mean field solutions and verifying that they are small. We do both in this subsection. In the case of Eq. (S1), the equations of motion are

$$\begin{aligned}\frac{d\langle \hat{a}\rangle}{dt} &= -\frac{\kappa}{2}\langle \hat{a}\rangle - ig\langle \hat{J}^-\rangle + \frac{\kappa\Omega_d}{4g}\\ \frac{d\langle \hat{J}^-\rangle}{dt} &= 2ig\langle \hat{a}\hat{J}_z\rangle \\ \frac{d\langle \hat{J}_z\rangle}{dt} &= -ig\left(\langle \hat{a}\hat{J}^+\rangle - \langle \hat{a}^\dagger \hat{J}^-\rangle\right)\end{aligned} \tag{S7}$$

and the mean field steady state solutions are obtained by factorizing the expectation values (e.g. $\langle \hat{a}\hat{J}_z\rangle \to \langle \hat{a}\rangle\langle \hat{J}_z\rangle$) and setting the time derivatives to 0. The superradiant phase is given by $\langle \hat{a}\rangle = \langle \hat{J}_x\rangle = 0$ as well as

$$\begin{aligned}\langle \hat{J}_y\rangle &= \frac{\Omega_d}{(4g^2N/\kappa)} \equiv \frac{N}{2}\sin\theta\\ \langle \hat{J}_z\rangle &= -\frac{N}{2}\cos\theta,\end{aligned} \tag{S8}$$

where the angle θ is measured from the $-z$ axis towards the $+y$ axis. The mean field solution describes a spin of length $N/2$ pointing at an angle θ in the zy plane. Since spin length is conserved, the relevant fluctuations occur in directions transverse to the mean field spin and behave like boson quadratures for N large (*67*). We can thus write

$$\begin{aligned}\hat{J}_x &= \sqrt{\frac{N}{2}}\hat{x} + O(N^{-1/2})\\ \hat{J}_y\cos\theta + \hat{J}_z\sin\theta &= -\sqrt{\frac{N}{2}}\hat{p} + O(N^{-1/2})\\ \hat{J}_y\sin\theta - \hat{J}_z\cos\theta &= \frac{N}{2} + O(N^0),\end{aligned} \tag{S9}$$

where $[\hat{x},\hat{p}] = i$ are a pair of auxiliary Holstein-Primakoff bosons. The first two equations describe the transverse fluctuations and the third describes the variable along the Bloch vector direction, whose fluctuations we neglect because they are smaller due to spin length conservation. Plugging this decomposition into Eq. (S1) and keeping only the leading order non-vanishing terms (in a $1/N$ expansion) yields

$$\frac{d\hat{\rho}}{dt} = -\frac{ig\sqrt{N}}{\sqrt{2}}\left[\hat{a}^\dagger(\hat{x}+i\hat{p}\cos\theta) + \hat{a}(\hat{x}-i\hat{p}\cos\theta), \hat{\rho}\right] + \kappa\left(\hat{a}\hat{\rho}\hat{a}^\dagger - \frac{\{\hat{a}^\dagger\hat{a},\hat{\rho}\}}{2}\right) + O(gN^0\hat{p}^3\hat{a}). \tag{S10}$$

At this level of approximation, it can be checked that the steady state $\hat{\rho}_{ss} = |ss\rangle\langle ss|$ is given by

$$\hat{a}|ss\rangle = (\hat{x} + i\hat{p}\cos\theta)\,|ss\rangle = 0. \tag{S11}$$

The cavity field is truly in the vacuum state while spin fluctuations along the $\hat{S}_x$ direction are squeezed

$$\langle \hat{J}_x^2 \rangle_{ss} \approx \frac{N}{2} \langle \hat{x}^2 \rangle_{ss} = \frac{N}{4} \cos\theta. \tag{S12}$$

These results are valid away from the transition point, when fluctuations in $\hat{p}$ are small [see neglected terms in Eq. (S10)]. This derivation is valid for $N \rightarrow \infty$ at fixed $g\sqrt{N}/\kappa$, but it didn't rely on any assumption about the actual value of $g\sqrt{N}/\kappa$, so the results should also be valid for Eq. (S2). In fact, the mean field spin direction Eq. (S8) and the spin fluctuations are also identical to the results for the master equation of cooperative resonance fluorescence Eq. (S2), the latter of which has been studied extensively in the literature (*21, 49, 68*).

We finalize this section with a comment pertaining to experimental implementations. The experiment we report in the main text is described by Eq. (S1). Alternatively, if the atoms were driven directly through the side of the cavity (instead of through the cavity mirrors) the experiment would be directly described by Eq. (S4). The atomic response would be identical but the cavity state would differ because now the superradiant phase ($\Omega_d < 2g^2N/\kappa$) would be populated by a large number of photons ($\langle \hat{b} \rangle = \Omega_d/2g$). Thus, the presence or absence of a macroscopically large cavity field is context dependent.

Normal phase The steady state solution to CRF Eq. (S2) can be written down analytically (*21*)

$$\hat{\rho} = \left(\frac{1}{\hat{J}^- + \frac{i\Omega_d}{4g^2/\kappa}} \right) \times \left(\frac{1}{\hat{J}^+ - \frac{i\Omega_d}{4g^2/\kappa}} \right). \tag{S13}$$

This formula is valid in both the superradiant and the normal phase, but in the normal phase ($\Omega > 2g^2N/\kappa$) this expression for the steady state can be re-interpreted as a probability distribution on the surface of the sphere. This is achieved by doing the replacements $\hat{J}^- \longrightarrow (N/2)\sin\theta e^{-i\phi}$, $\hat{J}_z \longrightarrow (N/2)\cos\theta$, leading to

$$P(\theta,\phi) \propto \left| \frac{1}{\frac{N}{2}\sin\theta e^{i\phi} + \frac{i\Omega_d}{4g^2/\kappa}} \right|^2 . \tag{S14}$$

Expectation values are then obtained by integration with respect to the measure $\sin\theta d\theta d\phi$. The steady state in this regime is mixed (*21*), with large fluctuations for the spin variables, and therefore it is not captured correctly by a mean-field treatment. Nevertheless, it is still captured by a classical

probability distribution (*21, 33*). Furthermore, the equilibration timescale to this steady state (which we call the "beyond mean-field" timescale) is $(4g^2/\kappa)^{-1}$, is N times longer than the equilibration timescales deep in the superradiant phase [$\sim (4g^2N/\kappa)^{-1}$]. The behaviour at times much shorter than the "beyond mean-field" timescale is instead characterized by persistent oscillations (for all initial conditions). These oscillations then decay due to beyond mean-field effects. The mean-field equations of motion capture accurately the oscillations but not their decay.

For Eq. (S6) there is no analytical solution to the steady state, so mathematically exact statements are not available. Nevertheless, we expect a similar phenomenology to hold (*57*). To begin with, there is no longer a stable steady state to the mean-field equations of motion. The remaining stationary mean-field solutions are neither stable nor unstable (i.e., they are centers), so that at times shorter than the "beyond mean-field" timescale we can expect persistent oscillations. Such dynamics is analogous to limit cycles found in (*57*), which disappear once beyond mean-field effect or spontaneous emission is considered. At longer times the dynamical noise coming from dissipation will induce diffusion between (and within) the oscillatory trajectories, leading to a mixed steady state in the same spirit as Eq. (S18).

Numerical simulations in the presence of inhomogeneous broadening

In this section we provide a mathematical description of the system that includes relevant technical imperfections and derive the full set of mean field equations used to construct the theoretical curves of Figs. 2, 3, 4 and 5 in the main text.

In the presence of inhomogeneous broadening of the atomic transition, the system evolves according to the following master equation

$$\frac{d\hat{\rho}}{dt} = -i\left[\hat{H}_{\text{tot}}, \hat{\rho}\right] + \kappa\,\mathcal{L}_c(\hat{\rho}) + \gamma\,\mathcal{L}_{se}(\hat{\rho}). \tag{S15}$$

The Hamiltonian is divided into three pieces characterizing the atoms, cavity and atom-light interaction:

$$\hat{H}_{\text{tot}} = \underbrace{\sum_{k=1}^{N}(\omega_a+\delta_k)\hat{s}_k^z}_{\text{atoms}} + \underbrace{\omega_c\hat{a}^\dagger\hat{a} - i\frac{\kappa\Omega_d}{4g_{\text{rms}}}(\hat{a}e^{i\omega_d t} - \hat{a}^\dagger e^{-i\omega_d t})}_{\text{cavity}} + \underbrace{\sum_{k=1}^{N} g_k(\hat{a}\hat{s}_k^+ + \hat{a}^\dagger\hat{s}_k^-)}_{\text{interaction}}, \tag{S16}$$

where $\hat{a}$ ($\hat{a}^\dagger$) are operators for the cavity mode, $\hat{s}_k^{z,\pm}$ are spin 1/2 operators for atom k, ω_a is the atomic transition frequency, ω_c is the cavity resonance frequency, g_k is the coupling of atom k

to the cavity mode and $g_{\rm rms}$ is the root-mean-square average of all the g_k. A 1D optical lattice along the cavity axis pins the position of the atoms in the longitudinal direction of the cavity. Confinement along the transverse direction is weaker and relies on the radially varying intensity of the optical lattice laser. As the atoms explore positions away from the peak intensity of the Gaussian beams, they feel different AC Stark shifts to the atomic transition frequency. This is encoded in the inhomogeneous detunings δ_k, which follow a distribution

$$P(\delta) = \left(\frac{U_0}{k_B T \delta_{\rm max}}\right)\left(\frac{\delta}{\delta_{\rm max}}\right)^{U_0/(k_B T)-1}, \qquad 0 < \delta < \delta_{\rm max}, \tag{S17}$$

where $U_0/\hbar = 2\pi \times 1.98$ MHz is the trap depth and $T = 15\mu$K is the temperature of the atoms ($U_0 = 6.34 k_B T$, where k_B is the Boltzmann constant), and the distribution is determined by the combined effect of the Gaussian profile of the laser beam and the thermal atomic distribution in the presence of the confining potential. The few atoms that are far away from the center of the spot feel almost no intensity and no frequency shift $\delta \approx 0$. However, most atoms are concentrated near the center and feel the maximum possible intensity and suffer a frequency shift $\delta_{\rm max} \approx 2\pi \times 125(25)$kHz, a number that is inferred from the experimental setup. The physical response of the system is determined mostly by the standard deviation of this distribution, which for $U_0 = 6.34 k_B T$ is about $0.11\delta_{\rm max} \approx 2\pi \times 14(3)$ kHz (roughly the same size as γ).

The experiment is calibrated in a way that puts the cavity on resonance with the average atomic transition frequency $\omega_c = \omega_a + \bar{\delta}$, where $\bar{\delta}$ is the average of Eq. (S17). The laser drive is then put on resonance with the cavity ($\omega_d = \omega_c$) so that in the rotating frame of the drive we have the Hamiltonian

$$\hat{H}' = \sum_{k=1}^{N} (\delta_k - \bar{\delta})\hat{s}_k^z - i\frac{\kappa\Omega_d}{4g_{\rm rms}}(\hat{a} - \hat{a}) + \sum_{k=1}^{N} g_k(\hat{a}\hat{s}_k^+ + \hat{a}^\dagger \hat{s}_k^-). \tag{S18}$$

As described in the Methods, in a standing wave cavity the coupling constants have the form $g_k = g_0 \cos(\phi_k)$, where $\phi_k = 2\pi k \lambda_l/\lambda_c$, $g_0 = 2\pi \times 10.8$ kHz is the single-photon Rabi frequency at an antinode of the cavity and $\lambda_{l/c}$ are the wavelengths of the lattice/cavity respectively. Since λ_l and λ_c are incommesurate, we can assume that ϕ_k is distributed uniformly in the interval $(0, 2\pi]$. In particular, the root-mean-square coupling $g_{\rm rms}$ is related to g_0 by $g_{\rm rms} = g_0/\sqrt{2}$. We'll express all quantities in terms of $g_{\rm rms}$ instead of g_0. In the main text we defined $g \equiv g_{\rm rms}$, but here in this Supplementary Material we will keep the rms subscript explicit wherever it appears.

The mean field equations of motion in the rotating frame of the drive are

$$
\begin{aligned}
\dot{\alpha} &= -\frac{\kappa}{2}\alpha - i\sqrt{N}\left(\frac{1}{N}\sum_k g_k s_k\right) + \frac{\kappa\Omega_d}{4g_{\text{rms}}\sqrt{N}} \\
\dot{s}_k &= 2ig_k\sqrt{N}\alpha z_k - \frac{\gamma}{2}s_k - i(\delta_k - \bar{\delta})s_k \\
\dot{z}_k &= -ig_k\sqrt{N}(\alpha s_k^* - \alpha^* s_k) - \gamma\left(z_k + \frac{1}{2}\right),
\end{aligned}
\tag{S19}
$$

where $\alpha = \langle\hat{a}\rangle/\sqrt{N}$, $s_k = \langle\hat{s}_k^-\rangle$ and $z_k = \langle\hat{s}_k^z\rangle$. The equation for α involves only a weighted average coherence

$$
\frac{1}{N}\sum_k g_k s_k \approx \int_0^{2\pi}\left(\sqrt{2}g_{\text{rms}}\cos\phi\right)\frac{d\phi}{2\pi}\int_0^{\delta_{\max}} P(\delta)s(\delta,\phi)\,d\delta, \tag{S20}
$$

where we have traded the index k for the pair of variables ϕ, δ and assumed that the coupling and detuning distributions are uncorrelated because they arise from independent physical effects. We find numerically that a grid of 20 values of ϕ and 40 values of δ is sufficient to get convergence in the superradiant phase. To prepare the steady state adiabatically, we use a time dependent drive $\Omega_d(t)$:

$$
\Omega_d(t) = \begin{cases} \Omega_d\left(\frac{t}{T_{\text{ramp}}}\right) & t < T_{\text{ramp}} \\ \Omega_d & T_{\text{ramp}} < t < T_{\text{hold}}. \end{cases} \tag{S21}
$$

The drive strength rises linearly from 0 to Ω_d during a time T_{ramp} and is then held fixed until a time T_{hold}. For the simulations, we use the following parameters

$$
\begin{aligned}
\kappa &= 2\pi \times 153 \text{ kHz} \\
\gamma &= 2\pi \times 7.5 \text{ kHz} \\
g_{\text{rms}} &= 2\pi \times 7.8 \text{ kHz} \\
\delta_{\max} &= 2\pi \times 125 \text{ kHz} \\
N &= 10^3 - 10^4
\end{aligned}
\tag{S22}
$$

The exact atom number N is taken directly from experimental measurements.

Finally, as pointed out in the Methods, the atomic inversion is measured via vaccuum Rabi splitting, which provides a weighted average

$$
\tilde{J}_z = N\frac{\sum_k g_k^2 z_k}{\sum_k g_k^2}, \tag{S23}
$$

and the normalization is chosen so that $\tilde{J}_z = -N/2$ when all the atoms are in their ground state. Our numerical simulations account for this weighting directly and the analytical results we will describe in the following sections will also focus on computing $\tilde{J}_z$.

Steady state behaviour

In this section we consider two broad steady state scenarios: (i) ideal CRF transition, with $\gamma = 0$ and $\delta = 0$, (ii) first order transition, including nonzero γ and δ_k. In a few cases, the mean field equations in Eq. (S19) can be solved analytically.

Continuous superradiant transition Here we set $\gamma = 0$ and $\delta_k = 0$ in Eq. (S19). In this situation there are many possible steady states (at the mean-field level) since the only requirement to get a steady state solution to Eq. (S8) with 0 intracavity field (and hence no atomic dynamics) is that $\sum_k g_k s_k = 0$. The relevant configuration for us is the one that we access through the drive, so we need to partly solve the dynamics. If all the atoms start from the ground state (south pole of Bloch sphere), then their individual Bloch vectors will lie on the yz plane, so they can be parameterized as $z_k = -(1/2)\cos\theta_k$ and $s = -(i/2)\sin\theta_k$. This parameterization describes a rotation towards the equator by an angle θ_k that is measured from $-z$ towards $+y$. Plugging this into Eq. (S19) leads to $\theta_k = g_k\sqrt{N}Q$, where $Q = \int_0^T 2\alpha\, dt$ is common to all the spins. The dynamics is thus reduced to two equations

$$\begin{aligned} \dot{\alpha} &= -\frac{\kappa}{2}\alpha - \sqrt{N}\left[\frac{1}{2N}\sum_k g_k \sin(g_k\sqrt{N}Q)\right] + \frac{\kappa\Omega_d}{4g_{\rm rms}\sqrt{N}} \\ \dot{Q} &= 2\alpha \end{aligned} \tag{S24}$$

There is now a unique steady state solution, characterized by $\alpha_{ss} = 0$ and a Q_{ss} that satisfies

$$\sqrt{N}\left[\frac{1}{2N}\sum_k g_k \sin(g_k Q_{ss})\right] = \frac{\kappa\Omega_d}{4g_{\rm rms}\sqrt{N}} \tag{S25}$$

We distinguish the two-following cases:

- *Uniform couplings ($g_k = g_{rms}$):* The steady state solution is determined by

$$\sin(g_{\rm rms}\sqrt{N}Q_{ss}) = \frac{\Omega_d}{2g_{\rm rms}^2 N/\kappa}, \tag{S26}$$

and a solution exists only if $\Omega_d < \Omega_c^h \equiv 2g_{\rm rms}^2 N/\kappa$. In this case the weighted inversion coincides with the true inversion of the system and satisfies

$$2\tilde{J}_z/N = -\sqrt{1 - \left(\frac{\Omega_d}{\Omega_c^h}\right)^2} \tag{S27}$$

- *Non-uniform couplings ($g_k = \sqrt{2} g_{rms} \cos\phi_k$):* The steady state is given by

$$J_1\left(\sqrt{2} g_{\rm rms}\sqrt{N} Q_{ss}\right) = \frac{\Omega_d}{\sqrt{2}\Omega_c^h}, \tag{S28}$$

where J_1 is a Bessel function and has a solution for Q_{ss} only when $\Omega_d < \Omega_c^{nh} = 0.82\Omega_c^h$. The weighted inversion is given by

$$2\tilde{J}_z/N = -\left[J_0\left(\sqrt{2} g_{\rm rms}\sqrt{N} Q_{ss}\right) - J_2\left(\sqrt{2} g_{\rm rms}\sqrt{N} Q_{ss}\right)\right] \tag{S29}$$

Numerical simulations that incorporate the short time effects of spontaneous emission indicate that spontaneous emission further shifts the transition point to $\Omega_c^{nh,*} \equiv 0.78\Omega_c^h$ [see Fig. S3(a)]. Inhomogeneous broadening of the atomic transition further shifts the transition point to $\Omega_c \equiv 0.70\Omega_c^h$, which is what is observed experimentally. Furthermore, once we rescale each curve by their respective critical point, we find that they fall on top of each other [Fig. S3(b)].

First order transition Here we include the effects of spontaneous emission γ and inhomogeneous broadening δ_k. The mean field steady state is then unique and can be obtained explicitly from Eq. (S19):

$$\begin{aligned} z_k &= -\frac{1}{2}\left\{1 + |\beta|^2\left[\frac{\eta_k^2}{1 + \frac{4(\delta_k - \bar{\delta})^2}{\gamma^2}}\right]\right\}^{-1} \\ s_k &= -i\beta\sqrt{\frac{1}{2}}\left[\frac{\eta_k}{1 + \frac{2i(\delta_k - \bar{\delta})}{\gamma}}\right]\left\{1 + |\beta|^2\left[\frac{\eta_k^2}{1 + \frac{4(\delta_k - \bar{\delta})^2}{\gamma^2}}\right]\right\}^{-1}, \end{aligned} \tag{S30}$$

which are expressed as functions of the normalized intracavity field

$$\beta = \alpha\left(\sqrt{\frac{8g_{\rm rms}^2 N}{\gamma^2}}\right), \tag{S31}$$

and of the normalized coupling $\eta_k = g_k/g_{\rm rms}$. The normalized field $|\beta|$ satisfies the equation

$$\sqrt{2}\beta\left[\left(\frac{\kappa\gamma}{4g_{\rm rms}^2 N}\right)+\left(\frac{1}{N}\sum_k\left[\frac{\eta_k^2}{1+\frac{2i(\delta_k-\bar{\delta})}{\gamma}}\right]\left\{1+|\beta|^2\left[\frac{\eta_k^2}{1+\frac{4(\delta_k-\bar{\delta})^2}{\gamma^2}}\right]\right\}^{-1}\right)\right]=\frac{\Omega_d}{\Omega_c^h}, \tag{S32}$$

which must be solved for $|\beta|$ as a function of Ω_d/Ω_c^h. Since $|\beta| \sim 1$ in the superradiant phase and we operate at a large collective cooperativity $NC_{\rm rms} = 4g_{\rm rms}^2 N/(\kappa\gamma)$, we neglect the first term on the left hand side. We then have the following cases (we also include a summary of the results in table S1):

- *Uniform couplings ($g_k = g_{rms}$) and no broadening ($\delta_k - \bar{\delta} = 0$):* the equation for the field $|\beta|$ becomes

$$\frac{\sqrt{2}\beta}{1+|\beta|^2}=\frac{\Omega_d}{\Omega_c^h}, \tag{S33}$$

which has a solution only when $\Omega_d < \Omega_c^h/\sqrt{2}$. In this uniform case, spontaneous emission transforms the second order transition at Ω_c^h to a first order transition at $\Omega_c^{h,se} \equiv \Omega_c^h/\sqrt{2}$. The weighted inversion can also be calculated, yielding

$$2\tilde{J}_z/N = -\frac{1}{2}\left[1+\sqrt{1-2\left(\frac{\Omega_d}{\Omega_c^h}\right)^2}\right]. \tag{S34}$$

When $\Omega_d = 0$ then $\tilde{J}_z = -N/2$ and when $\Omega_d = \Omega_c^h/\sqrt{2}$ then $\tilde{J}_z = -N/4$. For larger Ω_d, Eq. (S32) indicates that $\tilde{J}_z/N \sim (NC_{\rm rms})^{-2} \to 0$, so there is a jump from $-N/4$ to 0 at $\Omega_c^{h,se} = \Omega_c^h/\sqrt{2}$.

- *Non-uniform couplings ($g_k = \sqrt{2}g_{rms}\cos\phi_k$) and no broadening ($\delta_k - \bar{\delta} = 0$):* the steady state field in the superradiant phase is given by

$$\sqrt{2}\beta\int_0^{2\phi}\frac{d\phi}{2\pi}\left[\frac{2(\cos\phi)^2}{1+2|\beta|^2(\cos\phi)^2}\right]=\frac{\sqrt{2}\beta}{|\beta|^2}\left(1-\frac{1}{\sqrt{1+2|\beta|^2}}\right)=\frac{\Omega_d}{\Omega_c^h}, \tag{S35}$$

which has a solution when $\Omega_d < 0.6\Omega_c^h$. In this non-uniform coupling case, spontaneous emission transforms the second order transition at $\Omega_c^{nh} = 0.82\Omega_c^h$ to a first order transition at $\Omega_c^{nh,se} = 0.6\Omega_c^h$.

- *Non-uniform couplings ($g_k = \sqrt{2}g_{rms}\cos\phi_k$) and non-zero broadening ($\delta_k - \bar{\delta} \neq 0$):* This case describes the experimental conditions. The equation for the field $|\beta|$ can only be done

analytically to a certain extent

$$\sqrt{2}\beta\int P(\delta)\,d\delta\int_0^{2\pi}\frac{d\phi}{2\pi}\frac{2(\cos\phi)^2}{1+\frac{2i(\delta-\bar{\delta})}{\gamma}}\left\{1+\left[\frac{2|\beta|^2(\cos\phi)^2}{1+\frac{4(\delta_k-\bar{\delta})^2}{\gamma^2}}\right]\right\}^{-1}=\frac{\Omega_d}{\Omega_c^h}$$

$$\longrightarrow\frac{\sqrt{2}\beta}{|\beta|^2}\int P(\delta)\,d\delta\left[1-\frac{\sqrt{1+\frac{4(\delta-\bar{\delta})^2}{\gamma^2}}}{\sqrt{1+\frac{4(\delta-\bar{\delta})^2}{\gamma^2}+2|\beta|^2}}\right]\left[1-\frac{2i(\delta-\bar{\delta})}{\gamma}\right]=\frac{\Omega_d}{\Omega_c^h} \tag{S36}$$

The numerical solution of the previous equation using Eq. (S17) indicates that the first order transition is shifted. When $\delta_{\max} = 100\text{kHz}$, a solution exists only for $\Omega_d < 0.29\Omega_c^h = 0.41\Omega_c$. For $\delta_{\max} = 125\text{kHz}$, a solution exists only for $\Omega_d < 0.26\Omega_c^h = 0.37\Omega_c$. For $\delta_{\max} = 150\text{kHz}$, a solution only exists for $\Omega_d < 0.24\Omega_c^h = 0.33\Omega_c$. We've expressed the previous results also in terms of $\Omega_c = 0.71\Omega_c^h$, i.e. the experimentally determined critical point for the collective transition.

In Fig. S4(a) we show $\tilde{J}_z$ as a function of drive strength for the three analyzed cases. Furthermore, in Fig. S4(b) we compare these profiles against $\tilde{J}_z$ obtained via time evolution of Eq. (S19) for long but finite times. The time-dependent calculations do approach the steady state result, but the region around the transition jump takes a very long time to equilibrate.

Bistability In fact, optical bistability is expected in the system we have studied in the previous section titled "First order transition". This comes from the fact that Eq. (S37)

$$\sqrt{2}\beta\left[\left(\frac{\kappa\gamma}{4g_{\text{rms}}^2N}\right)+\left(\frac{1}{N}\sum_k\left[\frac{\eta_k^2}{1+\frac{2i(\delta_k-\bar{\delta})}{\gamma}}\right]\left\{1+|\beta|^2\left[\frac{\eta_k^2}{1+\frac{4(\delta_k-\bar{\delta})^2}{\gamma^2}}\right]\right\}^{-1}\right)\right]=\frac{\Omega_d}{\Omega_c^h}, \tag{S37}$$

has two solutions. One solution, characterized by $|\beta|\sim 1$ which we associated with the superradiant steady states in the previous section, corresponds to the "cooperative branch" in the bistability literature. There is another possible solution with $|\beta|\gg 1$. In this regime, Eq. (S37) acquires a universal form

$$\sqrt{2}\beta\left[\left(\frac{\kappa\gamma}{4g_{\text{rms}}^2N}\right)+\frac{1}{|\beta|^2}+O(|\beta|^{-4})\right]=\frac{\Omega_d}{\Omega_c^h}, \tag{S38}$$

Neglecting the $|\beta|^{-2}$ term inside the parentheses, we find that

$$\beta=\frac{\Omega_d}{\Omega_c^h}\left(\frac{2\sqrt{2}g_{\text{rms}}^2N}{\kappa\gamma}\right), \tag{S39}$$

which is the bare cavity response to the drive and self-consistently satisfies $|\beta| \gg 1$ whenever $g_{\rm rms}^2 N \gg \kappa\gamma$, as is the case for our system. This solution exists for

$$\frac{\Omega_d}{\Omega_c^h} \geq \frac{2}{\sqrt{4g_{\rm rms}^2 N/(\kappa\gamma)}}. \tag{S40}$$

This corresponds to the "high-transmission branch" of the bistable response (*35*), with small atomic inversion $z_k \sim |\beta|^{-2}$ and coherence $s_k \sim |\beta|^{-1}$. This shows that the high-transmission branch exists for drive strengths Ω_d much smaller than Ω_c^h. The first-order critical point that we describe in the main text should then be identified with the drive strength at which the system would jump to the "high-transmission" branch in a more standard bistability experiment where the drive strength is slowly ramped up.

Atom-cavity detuning In this subsection we explore the effects of an atom-cavity detuning on the second-order continuous transition. As described in the main text, we don't expect the profile of $\tilde{J}_z$ as a function of Ω_d/Ω_c^h to change substantially. We test this by modifying Eq. (S19) to include an atom-cavity detuning $\Delta_{ca} = \omega_c - \omega_a$:

$$\dot{\alpha} = -\frac{\kappa}{2}\alpha - i\Delta_{ca}\alpha - i\sqrt{N}\left(\frac{1}{N}\sum_k g_k s_k\right) + \frac{\kappa\Omega_d}{4g_{\rm rms}\sqrt{N}} \tag{S41}$$

and simulating the mean field equations of motion for $\Delta_{ca} = 0, \pm 2\kappa, \pm 5\kappa$. We show the results in Fig. S5, which corroborates our assertion that Δ_{ca} does not modify the transition point. The highest discrepancy is found at $\Delta_{ca} = -5\kappa$ close to the transition and arises from the finite evolution time.

Dynamical behaviour

In the experiment we also examined the dynamical behaviour of the system. For the second order transition, we explored the effect of a quench in the drive Ω_d. For the first order transition, we monitored $\tilde{J}_z$ as it slowly drifted due to spontaneous emission. In this section we provide a simplified theoretical analysis of both effects.

Short-time quenches If we begin with all $z_k = -1/2$ and $\alpha = 0$ and the drive Ω_d is suddenly turned on, the system has to adjust to the new steady state. This process is described by Eq. (S24) with the initial condition $z_k = -1/2$, $s_k = 0$ and $\alpha = 0$. Because the equations are nonlinear a

general solution is not possible, but we can analyze the behaviour close to the steady state. To do this, we linearize Eq. (S24), leading to

$$\begin{aligned} \delta\dot{\alpha} &= -\frac{\kappa}{2}\delta\alpha - \frac{\delta Q}{2}\left[\sum_k g_k^2\cos(g_k\sqrt{N}Q_{ss})\right] = -\frac{\kappa}{2}\delta\alpha + g_{\rm rms}^2\tilde{J}_z\delta Q \\ \delta\dot{Q} &= 2\delta\alpha, \end{aligned} \tag{S42}$$

where $\delta Q = Q - Q_{ss}$, $\delta\alpha = \alpha - \alpha_{ss}$ and $\tilde{J}_z$ is defined in Eq. (S29). The linearized equation has dynamical eigenvalues

$$-\frac{\kappa}{4} \pm i\sqrt{2g_{\rm rms}^2|\tilde{J}_z| - \frac{\kappa^2}{16}}. \tag{S43}$$

The approach to the steady state is thus characterized by oscillations with frequency $\sim \sqrt{2g_{\rm rms}^2|\tilde{J}_z|}$ and $1/e$ decay time $4/\kappa \approx 4\mu s$. This is illustrated in Fig. S6, which showcases oscillations and their decay. We also include curves with spontanteous emission and $\delta_{\rm max} = 2\pi \times 125$ kHz to investigate their effect on the short time dynamics. For the first few oscillations, they don't do much.

Long-time evolution due to spontaneous emission In this section, we analyze Eq. (S19) under the assumption that γ, $\sqrt{\text{Variance}(\delta_k)} \ll \kappa, g_{\rm rms}\sqrt{N}$ and in the regime where the system would equilibrate to the superradiant steady state if γ, δ_k were 0. As argued in the main text, the physics at intermediate times can be described in terms of the instantaneous cancellation between the drive and the self-radiated field of the atoms. Initially, this cancellation is almost perfect. However, spontaneous emission starts destroying the atomic coherence, so that the field established by the drive is marginally larger than the field generated by the radiating dipole. This field rotates the Bloch vector upward, seeking equilibration, and this process is repeated.

Because of the almost perfect equilibration, both $\sum_k g_k s_k$ (where $\eta_k = g_k/g_{\rm rms}$) and α should be almost constant. Furthermore, the intracavity field should be almost 0, but there will be a small correction because the cancellation between drive and self-radiated field is no longer be perfect. This remnant intracavity field can be obtained by enforcing that $\sum_k g_k s_k$ be constant in its equation of motion (enforcing that α be constant leads to the zeroth order result that $\alpha = 0$), leading to

$$\alpha = \frac{\gamma}{4ig_{\rm rms}\sqrt{N}\tilde{J}_z}\underbrace{\sum_k \eta_k\left[1 + \frac{2i(\delta_k - \bar{\delta})}{\gamma}\right]s_k}_{R}, \tag{S44}$$

where $\eta_k = g_k/g_{\rm rms}$ and we have defined R as indicated. This remnant field is small because $\gamma/g_{\rm rms}\sqrt{N}$ is small and so will induce slow dynamics. Replacing this expression for α in the spin equations of motion yields

$$\begin{aligned} \dot{s}_k &\approx \gamma\left[\frac{R}{2\tilde{J}_z}\eta_k z_k - \frac{s_k}{2}\left(1+\frac{2i(\delta_k-\bar{\delta})}{\gamma}\right)\right] \\ \dot{z}_k &\approx -\gamma\left[\frac{1}{4i\tilde{J}_z}\eta_k(Rs_k^* - R^* s_k) + z_k + \frac{1}{2}\right] \end{aligned} \tag{S45}$$

These equations conserve $\sum_k \eta_k g_k$, but $\tilde{J}_z$ slowly decreases in magnitude, in accord with our expectations. Depending on the value of Ω_d, the system may reach a steady state that sustains a macroscopic dipole moment. Otherwise, $\tilde{J}_z$ will continue drifting towards 0. Once this happens, the cavity gets populated by photons and the atoms start Rabi flopping.

To obtain an estimate of the time needed for $\tilde{J}_z$ to reach 0, we work now with the homogeneous system ($g_k = g_{\rm rms}$, $\delta_k = 0$). There is now a single equation for $\tilde{J}_z$

$$\frac{d\tilde{J}_z}{dt} = -\frac{\gamma N}{8\tilde{J}_z/N}\left(\frac{\Omega_d}{\Omega_c^h}\right)^2 - \gamma\left(\tilde{J}_z + \frac{N}{2}\right). \tag{S46}$$

The two terms in the previous equation can be given a direct physical interpretation. The first one is the Rabi rotation caused by the small remnant intracavity field given by Eq. (S44) with $\delta_k = 0$ and $g_k = g_{\rm rms}$. This can be seen more clearly by noting that the intracavity field is calculated self-consistently via

$$\alpha \approx \frac{\gamma N s}{4i g_{\rm rms}\sqrt{N}\tilde{J}_z} \tag{S47}$$

and hence the Rabi rotation term on the equation for the z component of the spin is given by

$$i g_{\rm rms}\sqrt{N}(\bar{\alpha}s - \alpha\bar{s}) = -\frac{\gamma}{2\tilde{J}_z/N}|s|^2 = -\frac{\gamma}{8\tilde{J}_z/N}\left(\frac{\Omega_d}{\Omega_c^h}\right)^2, \tag{S48}$$

where we are also replacing $s = -i\Omega_d/(2\Omega_c^h)$ since it is constant. This term pushes $\tilde{J}_z$ towards 0. The second term in Eq. (S46) accounts for the repumping of atoms into the ground state caused by spontaneous emission and pushes $\tilde{J}_z$ towards -1. When $\Omega_d < \Omega_c^h/\sqrt{2}$ the competition between these two terms leads to equilibration at a finite value of $\tilde{J}_z$. When $\Omega_c^h/\sqrt{2} < \Omega_d < \Omega_c^h$, then $\tilde{J}_z$ drift

slowly towards 0. The time is obtained by straightforward integration of Eq. (S46)

$$
\begin{aligned}
\gamma T &= -\int_{-\frac{2\tilde{J}_z^{ss}}{N}}^{0} \frac{x\,dx}{\frac{1}{2}\left(\frac{\Omega_d}{\Omega_c^h}\right)^2 + x\,(x+1)} \\
&= \frac{1}{2}\log\left(\frac{2(\Omega_c^h)^2 - \Omega_d^2 - 2\sqrt{(\Omega_c^h)^2 - \Omega_d^2}}{\Omega_d^2}\right) \\
&+ \frac{\Omega_c^h}{\sqrt{2\Omega_d^2 - (\Omega_c^h)^2}}\left[\arctan\left(\frac{2\sqrt{(\Omega_c^h)^2 - \Omega_d^2} - \Omega_c^h}{\sqrt{2\Omega_d^2 - (\Omega_c^h)^2}}\right) + \arctan\left(\frac{\Omega_c^h}{\sqrt{2\Omega_d^2 - (\Omega_c^h)^2}}\right)\right],
\end{aligned}
\tag{S49}
$$

where $\tilde{J}_z^{ss}$ is the steady state value given by Eq. (S27). When $\Omega_d \longrightarrow \Omega_c^{h,se} = \Omega_c^h/\sqrt{2}$, this time diverges as $\gamma T \sim \left(\frac{\Omega_d}{\Omega_c^{h,se}} - 1\right)^{-1/2}$. For $\Omega_d \approx 1.06\Omega_c^{h,se}$ we have that $T = 4.8\gamma^{-1}$, so the macroscopic dipole and the (almost) zero intracavity field are sustained for longer than a typical spontaneous emission $1/e$ time. For $\Omega_d = 1.13\Omega_c^{h,se}$, then $T \approx 2\gamma^{-1}$. In Fig. S6(b) We compare the effects of the simple model given by Eq. (S46) (with $\Omega_d = 0.75\Omega_c^h = 1.06\Omega_c^{h,se}$) and the full evolution given by Eq. (S19) (with $\Omega_d = 0.637\Omega_c^h = 1.06\Omega_c^{nh,se}$). The differing values of Ω_d were chosen so that the distance from Ω_d to the respective critical point is the same when measured in units of their respective critical drives. The curves match up when $\tilde{J}_z(t = \kappa^{-1})$ in Eq. (S46) is chosen to match the value of $\tilde{J}_z$ obtained after equilibration to the superradiant phase at a time κ^{-1}.

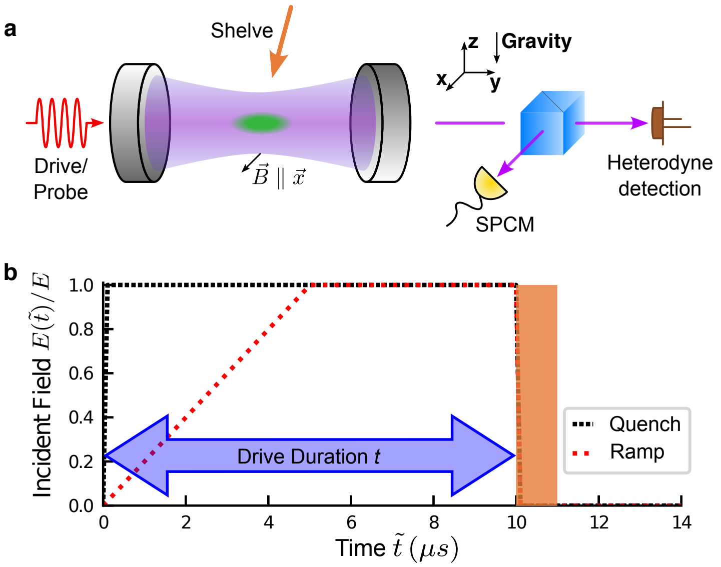

Figure S1: **Detailed experimental setup.** (**a**) ^{88}Sr atoms (green) are trapped inside a high finesse optical cavity. During the experiment, a horizontally polarized resonant 689 nm is sent towards the cavity. The intracavity field (purple) is monitored by directing 50% of the cavity transmission to a balanced heterodyne detection (brown). At the end of the drive duration, we shine a $\hat{y}$-polarized 688 nm beam from above (orange), resonant with the $^3P_1 - ^3S_1$ transition to optically pump the atoms in the excited state $|^3\mathrm{P}_1, m_J = 0\rangle$ to the metastable states $^3\mathrm{P}_0$ and $^3\mathrm{P}_2$, a procedure which we call "shelving" in the main text. We send weak $\hat{x}$-polarized 689 nm probe light before and after the drive and detect 50% of the cavity transmission on a single-photon counting module (SPCM) to perform a vacuum-Rabi splitting (VRS) measurement to count atom numbers in the ground state $|^1\mathrm{S}_0\rangle$. (**b**) An example of the temporal profile of the incident field for a 10 μs drive duration when ramping (red) and quenching (black) the drive. The y-axis is normalized to the maximum incident field E applied within a single shot. In both cases, the drive duration t is defined as the period from when the drive is initially turned on to when it is suddenly turned off. The orange shaded area indicates when the shelving of the excited state is performed.

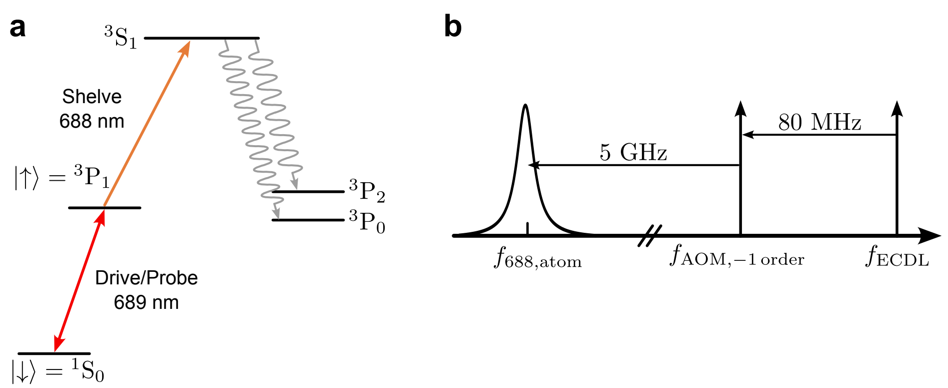

Figure S2: **Detailed experimental setup.** (**a**) Strontium level diagram. (**b**) 688 laser frequency shifting diagram. 50 ns before the 689 nm drive is turned off, we modulate a 688 nm AOM to turn on a -1st order sideband. Simultaneously with turning off the 689 nm drive, we turn on the 5 GHz sideband using a fiber EOM to generate a 688 nm tone resonant with the $^3P_1 - ^3S_1$ transition.

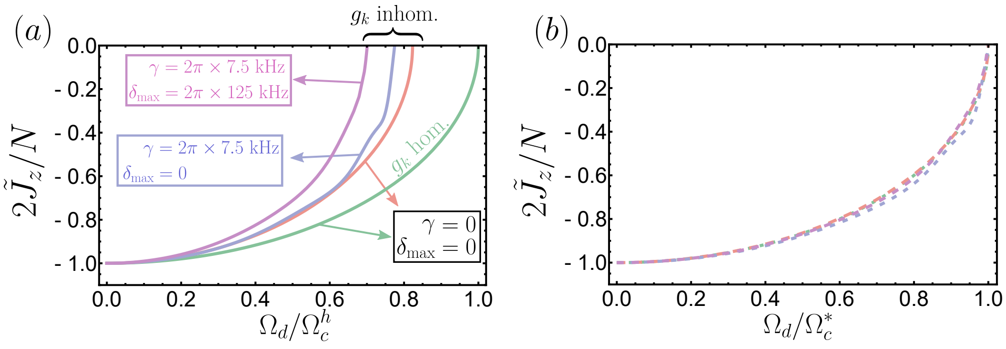

Figure S3: **Inversion $\tilde{J}_z$ as a function of drive strength Ω_d for the second-order transition.** (**a**) Inversion $\tilde{J}_z$ as a function of Ω_d/Ω_c^h for four different cases: homogeneous couplings with $\gamma = 0$ and $\delta_k = 0$ (green, analytical steady state), inhomogeneous couplings with $\gamma = 0$ and $\delta_k = 0$ (red, analytical steady state), inhomogenoeus couplings with $\gamma \neq 0$ and $\delta_k = 0$ (blue, numerical solution with $T_{\text{hold}} = 9.3\mu$s with $T_{\text{ramp}} = 5\mu$s), and inhomogeneous couplings with $\gamma \neq 0$ and $\delta_k = 0$ (purple, numerical solution with $T_{\text{hold}} = 9.3\mu$s with $T_{\text{ramp}} = 5\mu$s). (**b**) Same as panel (a) but after rescaling Ω_d by each respective critical drive: Ω_c^h (green), $\Omega_c^{nh} = 0.82\Omega_c^h$ (red), $\Omega_c^{nh,*} = 0.78\Omega_c^h$ (blue) and $\Omega_c = 0.70\Omega_c^h$ (purple). The Ω_c^* in the horizontal axis label represents each of these different transition frequencies.

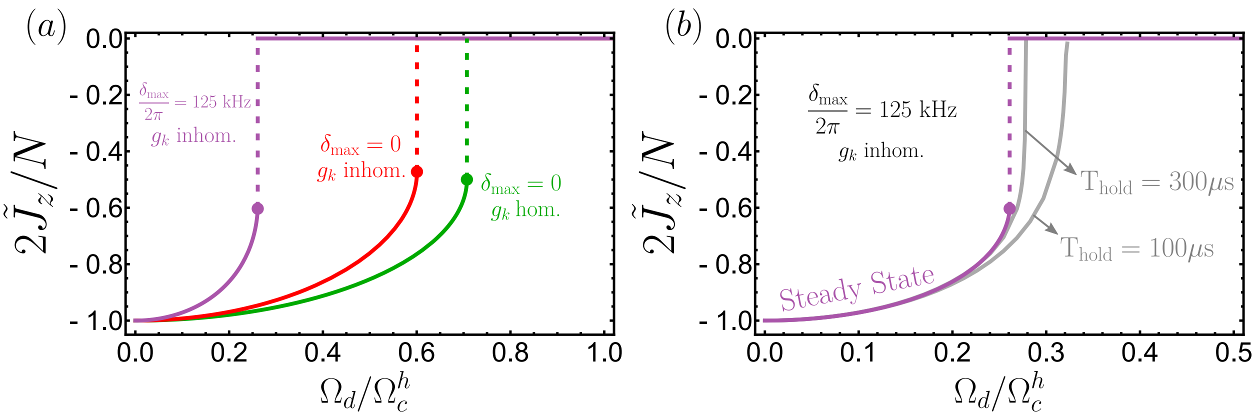

Figure S4: **Inversion $\tilde{J}_z$ as a function of drive strength Ω_d for the first-order transition.** (**a**) We show the long-time steady state weighted inversion $\tilde{J}_z$ as a function of Ω_d/Ω_c^h for three different scenarios: homogeneous couplings and no broadening (green), inhomogeneous couplings and no broadening (red), and inhomogeneous couplings with broadening (purple, $\delta_{\max} = 2\pi \times 125$ kHz). All curves incorporate the effects of spontaneous emission. (**b**) Comparison between the steady state profile for $\delta_{\max} = 2\pi \times 125$ kHz and time evolution for $T_{\text{hold}} = 100\mu$s, 300μs.

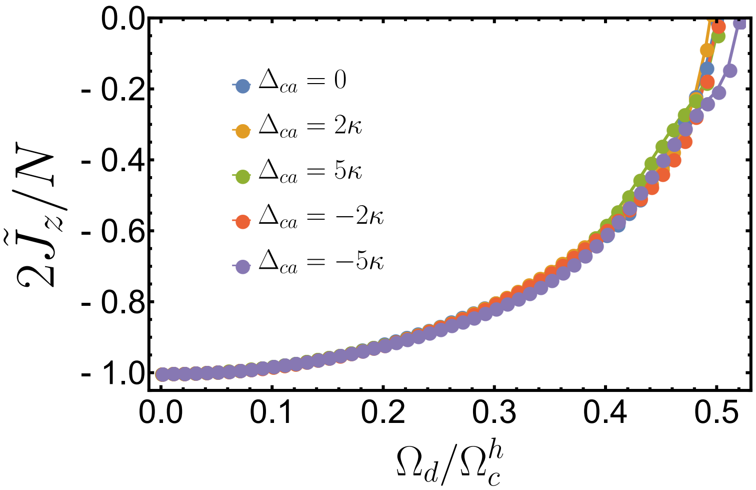

Figure S5: **Inversion $\tilde{J}_z$ as a function of drive strength Ω_d for different cavity detunings Δ_{ca}.** Inversion $\tilde{J}_z$ as a function of normalized Rabi frequency Ω_d/Ω_c^h for different values of Δ_{ca}. Simulations were done using a ramp time $T_{\text{ramp}} = 5\mu$s and total evolution time $T_{\text{hold}} = 9.3\mu$s in the presence of spontaneous emission and inhomogeneous broadening.

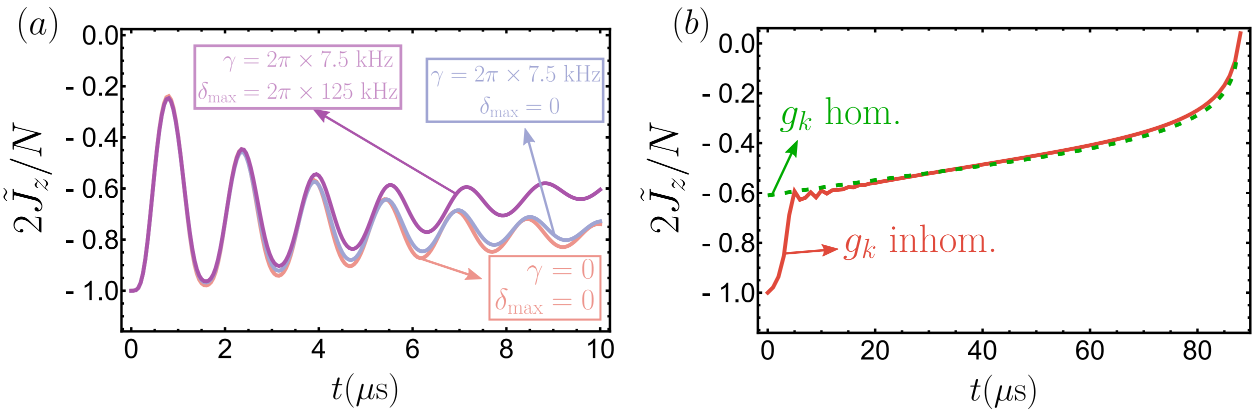

Figure S6: **Effects of inhomogenous coupling and spontaneous emission on dynamics of $\tilde{J}_z$.** (**a**) Short time dynamics of $\tilde{J}_z$ after a quench as a function of time for $N = 10^4$ and $\Omega = 0.52\Omega_c^h$. All curves include inhomogeneous couplings and show the evolution with $\gamma = \delta_{\max} = 0$ (red), $\gamma \neq 0$ and $\delta_{\max} = 0$ (blue), and $\gamma \neq 0$ and $\delta_{\max} = 2\pi \times 125$ kHz (purple). (**b**) Long time dynamics of $\tilde{J}_z$ in the presence of spontaneous emission using Eq. (S19) (red, $\Omega_d = 0.64\Omega_c^h$) and Eq. (S46) (dashed green, $\Omega_d = 0.75\Omega_c^h$) assuming no broadening ($\delta_{\max} = 0$).

Table S1: Critical point under different conditions.

	Uniform g_k	Non-uniform g_k	Non-uniform g_k
	$\delta_{\max} = 0$	$\delta_{\max} = 0$	$\delta_{\max} = 2\pi \times 125$ kHz
Continuous transition	Ω_c^h	$\Omega_c^{nh} = 0.82\Omega_c^h$	$\Omega_c = 0.701\Omega_c^h$ (includes γ)
First-order transition	$\Omega_c^{h,se} = 0.71\Omega_c^h$	$\Omega_c^{nh,se} = 0.6\Omega_c^h$	$0.26\Omega_c^h$

REFERENCES AND NOTES

1. I. M. Georgescu, S. Ashhab, F. Nori, Quantum simulation. *Rev. Mod. Phys.* **86**, 153–185 (2014).

2. E. Altman, K. R. Brown, G. Carleo, L. D. Carr, E. Demler, C. Chin, B. DeMarco, S. E. Economou, M. A. Eriksson, K. M. C. Fu, M. Greiner, K. R. A. Hazzard, R. G. Hulet, A. J. Kollár, B. L. Lev, M. D. Lukin, R. Ma, X. Mi, S. Misra, C. Monroe, K. Murch, Z. Nazario, K. K. Ni, A. C. Potter, P. Roushan, M. Saffman, M. Schleier-Smith, I. Siddiqi, R. Simmonds, M. Singh, I. B. Spielman, K. Temme, D. S. Weiss, J. Vučković, V. Vuletić, J. Ye, M. Zwierlein, Quantum simulators: Architectures and opportunities. *PRX Quantum* **2**, 017003 (2021).

3. A. J. Daley, Twenty-five years of analogue quantum simulation. *Nat. Rev. Phys.* **5**, 702–703 (2023).

4. K. Bharti, A. Cervera-Lierta, T. H. Kyaw, T. Haug, S. Alperin-Lea, A. Anand, M. Degroote, H. Heimonen, J. S. Kottmann, T. Menke, W.-K. Mok, S. Sim, L.-C. Kwek, A. Aspuru-Guzik, Noisy intermediate-scale quantum algorithms. *Rev. Mod. Phys.* **94**, 015004 (2022).

5. J. Ye, P. Zoller, Essay: Quantum sensing with atomic, molecular, and optical platforms for fundamental physics. *Phys. Rev. Lett.* **132**, 190001 (2024).

6. C. Couteau, S. Barz, T. Durt, T. Gerrits, J. Huwer, R. Prevedel, J. Rarity, A. Shields, G. Weihs, Applications of single photons to quantum communication and computing. *Nat. Rev. Phys.* **5**, 326–338 (2023).

7. R. H. Dicke, Coherence in spontaneous radiation processes. *Phys. Rev.* **93**, 99 (1954).

8. M. Gross, S. Haroche, Superradiance: An essay on the theory of collective spontaneous emission. *Phys. Rep.* **93**, 301–396 (1982).

9. J. G. Bohnet, Z. Chen, J. M. Weiner, D. Meiser, M. J. Holland, J. K. Thompson, A steady-state superradiant laser with less than one intracavity photon. *Nature* **484**, 78–81 (2012).

10. M. A. Norcia, M. N. Winchester, J. R. K. Cline, J. K. Thompson, Superradiance on the millihertz linewidth strontium clock transition. *Sci. Adv.* **2**, e1601231 (2016).

11. S. Inouye, A. P. Chikkatur, D. M. Stamper-Kurn, J. Stenger, D. E. Pritchard, W. Ketterle, Superradiant Rayleigh scattering from a Bose-Einstein condensate. *Science* **285**, 571–574 (1999).

12. A. Goban, C.-L. Hung, J. D. Hood, S.-P. Yu, J. A. Muniz, O. Painter, H. J. Kimble, Superradiance for atoms trapped along a photonic crystal waveguide. *Phys. Rev. Lett.* **115**, 063601 (2015).

13. C. Liedl, F. Tebbenjohanns, C. Bach, S. Pucher, A. Rauschenbeutel, P. Schneeweiss, Observation of superradiant bursts in a cascaded quantum system. *Phys. Rev. X* **14**, 011020 (2024).

14. M. Fitzpatrick, N. M. Sundaresan, A. C. Y. Li, J. Koch, A. A. Houck, Observation of a dissipative phase transition in a one-dimensional circuit QED lattice. *Phys. Rev. X* **7**, 011016 (2017).

15. M. Lei, R. Fukumori, J. Rochman, B. Zhu, M. Endres, J. Choi, A. Faraon, Many-body cavity quantum electrodynamics with driven inhomogeneous emitters. *Nature* **617**, 271–276 (2023).

16. W. Kersten, N. de Zordo, O. Diekmann, T. Reiter, M. Zens, A. N. Kanagin, S. Rotter, J. Schmiedmayer, A. Angerer, Triggered superradiance and spin inversion storage in a hybrid quantum system. *Phys. Rev. Lett.* **131**, 043601 (2023).

17. M. A. Norcia, J. R. K. Cline, J. A. Muniz, J. M. Robinson, R. B. Hutson, A. Goban, G. E. Marti, J. Ye, J. K. Thompson, Frequency measurements of superradiance from the strontium clock transition. *Phys. Rev. X* **8**, 021036 (2018).

18. S. L. Kristensen, E. Bohr, J. Robinson-Tait, T. Zelevinsky, J. W. Thomsen, J. H. Müller, Subnatural linewidth superradiant lasing with cold ^{88}Sr atoms. *Phys. Rev. Lett.* **130**, 223402 (2023).

19. I. R. Senitzky, Interaction between a nonlinear oscillator and a radiation field. *Phys. Rev. A* **6**, 1175–1196 (1972).

20. P. Drummond, H. Carmichael, Volterra cycles and the cooperative fluorescence critical point. *Opt. Commun.* **27**, 160–164 (1978).

21. H. J. Carmichael, Analytical and numerical results for the steady state in cooperative resonance fluorescence. *J. Phys. B: Atom. Mol. Phys.* **13**, 3551–3575 (1980).

22. K. Hepp, E. H. Lieb, Equilibrium statistical mechanics of matter interacting with the quantized radiation field. *Phys. Rev. A* **8**, 2517–2525 (1973).

23. F. Dimer, B. Estienne, A. S. Parkins, H. J. Carmichael, Proposed realization of the Dicke-model quantum phase transition in an optical cavity QED system. *Phys. Rev. A* **75**, 013804 (2007).

24. K. Baumann, C. Guerlin, F. Brennecke, T. Esslinger, Dicke quantum phase transition with a superfluid gas in an optical cavity. *Nature* **464**, 1301–1306 (2010).

25. R. M. Kroeze, Y. Guo, V. D. Vaidya, J. Keeling, B. L. Lev, Spinor self-ordering of a quantum gas in a cavity. *Phys. Rev. Lett.* **121**, 163601 (2018).

26. J. Klinder, H. Keßler, M. Wolke, L. Mathey, A. Hemmerich, Dynamical phase transition in the open Dicke model. *Proc. Natl. Acad. Sci. U.S.A.* **112**, 3290–3295 (2015).

27. Z. Zhiqiang, C. H. Lee, R. Kumar, K. J. Arnold, S. J. Masson, A. S. Parkins, M. D. Barrett, Nonequilibrium phase transition in a spin-1 Dicke model. *Optica* **4**, 424–429 (2017).

28. F. Ferri, R. Rosa-Medina, F. Finger, N. Dogra, M. Soriente, O. Zilberberg, T. Donner, T. Esslinger, Emerging dissipative phases in a superradiant quantum gas with tunable decay. *Phys. Rev. X* **11**, 041046 (2021).

29. A. Safavi-Naini, R. J. Lewis-Swan, J. G. Bohnet, M. Gärttner, K. A. Gilmore, J. E. Jordan, J. Cohn, J. K. Freericks, A. M. Rey, J. J. Bollinger, Verification of a many-ion simulator of the

Dicke model through slow quenches across a phase transition. *Phys. Rev. Lett.* **121**, 040503 (2018).

30. D. Meiser, J. Ye, D. R. Carlson, M. J. Holland, Prospects for a millihertz-linewidth laser. *Phys. Rev. Lett.* **102**, 163601 (2009).

31. G. Ferioli, A. Glicenstein, I. Ferrier-Barbut, A. Browaeys, A non-equilibrium superradiant phase transition in free space. *Nat. Phys.* **19**, 1345–1349 (2023).

32. D. Goncalves, L. Bombieri, G. Ferioli, S. Pancaldi, I. Ferrier-Barbut, A. Browaeys, E. Shahmoon, D. Chang, Driven-dissipative phase separation in free-space atomic ensembles. *PRXQuantum* **6**, 020303 (2025).

33. S. Agarwal, E. Chaparro, D. Barberena, A. P. Orioli, G. Ferioli, S. Pancaldi, I. Ferrier-Barbut, A. Browaeys, A. Rey, Directional superradiance in a driven ultracold atomic gas in free space. *PRX Quantum* **5**, 040335 (2024).

34. J. Ruostekoski, Superradiant phase transition in a large interacting driven atomic ensemble in free space. *Optica Quantum* **3**, 15–21 (2025).

35. R. Bonifacio, L. A. Lugiato, Optical bistability and cooperative effects in resonance fluorescence. *Phys. Rev. A* **18**, 1129–1144 (1978).

36. A. T. Rosenberger, L. A. Orozco, H. J. Kimble, Observation of absorptive bistability with two-level atoms in a ring cavity. *Phys. Rev. A* **28**, 2569–2572 (1983).

37. G. Rempe, R. J. Thompson, R. J. Brecha, W. D. Lee, H. J. Kimble, Optical bistability and photon statistics in cavity quantum electrodynamics. *Phys. Rev. Lett.* **67**, 1727–1730 (1991).

38. J. Gripp, S. L. Mielke, L. A. Orozco, H. J. Carmichael, Anharmonicity of the vacuum Rabi peaks in a many-atom system. *Phys. Rev. A* **54**, R3746–R3749 (1996).

39. D. Rivero, C. A. P. Jr, G. H. de França, R. C. Teixeira, S. Slama, P. W. Courteille, Quantum resonant optical bistability with a narrow atomic transition: bistability phase diagram in the bad cavity regime. *New J. Phys.* **25**, 093053 (2023).

40. B. Buča, T. Prosen, A note on symmetry reductions of the Lindblad equation: Transport in constrained open spin chains. *New J. Phys.* **14**, 073007 (2012).

41. V. V. Albert, L. Jiang, Symmetries and conserved quantities in Lindblad master equations. *Phys. Rev. A* **89**, 022118 (2014).

42. D. Roberts, A. A. Clerk, Exact solution of the infinite-range dissipative transverse-field Ising model. *Phys. Rev. Lett.* **131**, 190403 (2023).

43. J. T. Young, E. Chaparro, A. Piñeiro Orioli, J. K. Thompson, A. M. Rey, Engineering one axis twisting via a dissipative berry phase using strong symmetries. *Phys. Rev. Lett.* **134**, 040801 (2025).

44. M. J. Martin, D. Meiser, J. W. Thomsen, J. Ye, M. J. Holland, Extreme nonlinear response of ultranarrow optical transitions in cavity QED for laser stabilization. *Phys. Rev. A* **84**, 063813 (2011).

45. D. Barberena, R. J. Lewis-Swan, A. M. Rey, J. K. Thompson, Ultra narrow linewidth frequency reference via measurement and feedback. *C. R. Phys.* **24**, 55–68 (2023).

46. M. A. Norcia, J. K. Thompson, Strong coupling on a forbidden transition in strontium and nondestructive atom counting. *Phys. Rev. A* **93**, 023804 (2016).

47. D. F. Walls, P. D. Drummond, S. S. Hassan, H. J. Carmichael, Non-equilibrium phase transitions in cooperative atomic systems. *Prog. Theor. Phys.* **64**, 307–320 (1978).

48. O. Somech, Y. Shimshi, E. Shahmoon, Heisenberg-Langevin approach to driven superradiance. *Phys. Rev. A* **108**, 023725 (2023).

49. D. Barberena, A. M. Rey, Critical steady states of all-to-all squeezed and driven superradiance: An analytic approach. *Phys. Rev. A* **109**, 013709 (2024).

50. M. G. Raizen, R. J. Thompson, R. J. Brecha, H. J. Kimble, H. J. Carmichael, Normal-mode splitting and linewidth averaging for two-state atoms in an optical cavity. *Phys. Rev. Lett.* **63**, 240–243 (1989).

51. D. Barberena, R. J. Lewis-Swan, J. K. Thompson, A. M. Rey, Driven-dissipative quantum dynamics in ultra-long-lived dipoles in an optical cavity. *Phys. Rev. A* **99**, 053411 (2019).

52. R. Bonifacio, L. Lugiato, Cooperative effects and bistability for resonance fluorescence. *Opt. Commun.* **19**, 172–176 (1976).

53. N. Leppenen, E. Shahmoon, Quantum bistability at the interplay between collective and individual decay. arXiv:2404.02134 [quant-ph] (2024).

54. M. A. Norcia, J. K. Thompson, Cold-strontium laser in the superradiant crossover regime. *Phys. Rev. X* **6**, 011025 (2016).

55. D. J. Young, A. Chu, E. Y. Song, D. Barberena, D. Wellnitz, Z. Niu, V. M. Schäfer, R. J. Lewis-Swan, A. M. Rey, J. K. Thompson, Observing dynamical phases of BCS superconductors in a cavity QED simulator. *Nature* **625** (7996), 679–684 (2024).

56. J. A. Muniz, D. Barberena, R. J. Lewis-Swan, D. J. Young, J. R. K. Cline, A. M. Rey, J. K. Thompson, Exploring dynamical phase transitions with cold atoms in an optical cavity. *Nature* **580**, 602–607 (2020).

57. R. Mattes, I. Lesanovsky, F. Carollo, Entangled time-crystal phase in an open quantum light-matter system. *Phys. Rev. A* **108**, 062216 (2023).

58. M. A. Norcia, R. J. Lewis-Swan, J. R. K. Cline, B. Zhu, A. M. Rey, J. K. Thompson, Cavity-mediated collective spin-exchange interactions in a strontium superradiant laser. *Science* **361**, 259–262 (2018).

59. T. E. Lee, C.-K. Chan, S. F. Yelin, Dissipative phase transitions: Independent versus collective decay and spin squeezing. *Phys. Rev. A* **90**, 052109 (2014).

60. E. Pedrozo-Peñafiel, S. Colombo, C. Shu, A. F. Adiyatullin, Z. Li, E. Mendez, B. Braverman, A. Kawasaki, D. Akamatsu, Y. Xiao, V. Vuletić, Entanglement on an optical atomic-clock transition. *Nature* **588**, 414–418 (2020).

61. J. M. Robinson, M. Miklos, Y. M. Tso, C. J. Kennedy, T. Bothwell, D. Kedar, J. K. Thompson, J. Ye, Direct comparison of two spin-squeezed optical clock ensembles at the 10^{-17} level. *Nat. Phys.* **20**, 208–213 (2024).

62. D. A. Ivanov, T. Y. Ivanova, S. F. Caballero-Benitez, I. B. Mekhov, Feedback-induced quantum phase transitions using weak measurements. *Phys. Rev. Lett.* **124**, 010603 (2020).

63. G. Passarelli, X. Turkeshi, A. Russomanno, P. Lucignano, M. Schirò, R. Fazio, Many-body dynamics in monitored atomic gases without postselection barrier. *Phys. Rev. Lett.* **132**, 163401 (2024).

64. T. Ido, H. Katori, Recoil-free spectroscopy of neutral Sr atoms in the lamb-dicke regime. *Phys. Rev. Lett.* **91**, 053001 (2003).

65. R. J. Thompson, G. Rempe, H. J. Kimble, Observation of normal-mode splitting for an atom in an optical cavity. *Phys. Rev. Lett.* **68**, 1132–1135 (1992).

66. R. Bonifacio, P. Schwendimann, F. Haake, Quantum statistical theory of superradiance. *Phys. Rev. A* **4**, 302–313 (1971).

67. T. Holstein, H. Primakoff, Field dependence of the intrinsic domain magnetization of a ferromagnet. *Phys. Rev.* **58**, 1098–1113 (1940).

68. J. Hannukainen, J. Larson, Dissipation-driven quantum phase transitions and symmetry breaking. *Phys. Rev. A* **98**, 042113 (2018).
